# Impact of Lower-Volume Training on Physical Fitness Adaptations in Team Sports Players: A Systematic Review and Meta-analysis

**DOI:** 10.1186/s40798-024-00808-3

**Published:** 2025-01-20

**Authors:** Filipe Manuel Clemente, Rodrigo Ramirez-Campillo, Jason Moran, Piotr Zmijewski, Rui Miguel Silva, Morten Bredsgaard Randers

**Affiliations:** 1 Escola Superior Desporto e Lazer, Instituto Politécnico de Viana do Castelo, Rua Escola Industrial e Comercial de Nun’Álvares, 4900-347 Viana do Castelo, Portugal; 2 Sport Physical Activity and Health Research and Innovation Center, Viana do Castelo, Portugal; 3 Gdansk University of Physical Education and Sport, 80-336 Gdańsk, Poland; 4 Exercise and Rehabilitation Sciences Institute, School of Physical Therapy, Faculty of Rehabilitation Sciences, Universidad Andres Bello, Santiago, Chile; 5 School of Sport, Rehabilitation and Exercise Sciences, University of Essex, Colchester, Essex, UK; 6 Jozef Pilsudski University of Physical Education in Warsaw, Warsaw, Poland; 7 Department of Sports Science and Clinical Biomechanics, SDU Sport and Health Sciences Cluster, University of Southern Denmark, Odense, Denmark

**Keywords:** Team sports, Sports training, Training methodology, Training load

## Abstract

**Background:**

A small number of reviews have explored lower- versus higher-volume training in non-athletes, but the growing challenge of congested schedules in team sports highlights the need to synthesize evidence specific to team sport athletes. Thus, the objectives of this systematic review with meta-analysis are twofold: (i) to summarize the primary physiological and physical fitness outcomes of lower-volume versus higher-volume training interventions in team sports players; and (ii) to compare the effects of lower-volume training with higher, considering the training modalities used.

**Methods:**

We conducted searches across key databases, including PubMed, Scopus, SPORTDiscus, and Web of Science. We included team sports players with at least a trained or developmental level, focusing on studies comparing different training volumes (lower vs higher) within the same research. Lower volume training was defined in comparison to another load, emphasizing smaller training volume in terms of repetitions, duration, or frequency. The studies had to examine key physical performance adaptations and use two-arm or multi-arm designs. Methodological assessments of the included studies were performed using the Rob2 and ROBINS-I instruments, with evidence certainty evaluated through GRADE.

**Results:**

The initial search yielded 5,188 records, with 17 articles deemed eligible for the review. There was a non-significant trend favoring the higher-volume training group over the lower-volume group in resistance-based training when considering all pooled physical fitness outcomes (effect size − 0.05, 95% CI − 0.19 to 0.09, *p* = 0.506, *I*^2^ = 0.0%). A meta-analysis was not conducted for aerobic-based training due to only two studies being available, with one showing that lower volume training improved maximal oxygen uptake by 3.8% compared to 1.3% for higher volume, while the other indicated that lower training volumes enhanced performance by 1.6% versus 0.8%. The evidence certainty for physical performance outcomes was very low.

**Conclusions:**

In newly introduced resistance training, lower volumes—regardless of repetitions or frequency—can achieve similar fitness gains to higher volumes. More pronounced tapering also appears more effective for supercompensation. However, the variability in study designs and training methods makes it difficult to establish a clear minimal dose. The main contribution of this review is mapping current research, providing a foundation for future studies and training optimization.

**Supplementary Information:**

The online version contains supplementary material available at 10.1186/s40798-024-00808-3.

## Key Points

- Lower-volume resistance training—whether by halving the number of repetitions in sessions held at least twice a week or by reducing the training frequency by half—produces similar positive adaptations in the physical performance of team sport athletes compared to higher-volume training.
- In running-based and mixed-based training interventions, specifically in tapering strategies, reducing volume while maintaining intensity seems to yield a more favorable response than employing a higher volume.
- In tapering strategies, particularly concerning training load in field settings, more pronounced reductions in volume seem to enhance supercompensation. However, caution is warranted in interpreting this evidence, as the observed tendency could not be confirmed through meta-analytical approaches.

**Supplementary Information:**

The online version contains supplementary material available at 10.1186/s40798-024-00808-3.

## Background

The challenge of delivering effective strength and conditioning training that promotes positive adaptations without increasing residual fatigue in team sport athletes—who often face congested schedules—has been increasingly studied through comparisons of lower and higher training volumes (manipulating duration, repetitions, and/or frequency) in both resistance and aerobic training, as well as in contexts involving reduced overall training volume in field training [1]. A prominent factor contributing to this pertinence is the mounting prevalence of congested fixtures, leading to tightly packed competition schedules [2]. In such scenarios, the window for implementing targeted loads becomes considerably constrained [3]. Lower-volume training, characterized by fewer repetitions, shorter training duration, and/or fewer weekly sessions compared to normal or higher volumes, emerges as a possible strategy to provide adequate training stimuli while minimizing the onset of concurrent effects [4–6]. Prescribing excessive resistance and aerobic training alongside field practice can lead to residual fatigue [7] and impair metabolic recovery [8]. This overload may compromise physical readiness and, in more severe cases, result in decreased sports performance [9].

The notion of “volume” can be succinctly characterized as the outcome arising from the interplay of exercise intensity, duration, and frequency [10]. This signifies that modifications in the interrelation among exercise intensity, duration, or frequency can induce fluctuations in the resultant volume [11]. However, the classification of a given training volume as either “low” or “high” depends on contextual considerations, particularly considering the distinctive attributes of the sport, the nature of the training type and regimen, and the disjunction between anticipated and actual training loads [12]. Consequently, the endeavor to precisely delineate the confines of a “low-volume” paradigm presents a formidable task. In contrast, juxtaposing lower and higher volumes is more straightforward, as it entails a comparison embedded within a specifically defined framework.

For instance, in resistance training, one can compare lower versus higher training volumes by assessing total repetitions (e.g., 100 repetitions per week for lower volume vs 216 for higher volume) [71] or training frequency (e.g., one session per week for lower volume vs two sessions per week for higher volume, effectively doubling the volume) [5]. The aim is to provide a reduced volume through fewer repetitions per session or a lower overall weekly total. The same principles apply to aerobic training, where total training minutes per week can be adjusted by manipulating either the duration of each session or the cumulative minutes across multiple sessions [18].

Furthermore, in team sports, the manipulation of training volume can vary significantly based on the specifics of the sport. For example, in a team that competes only on weekends, reducing three high-intensity interval training sessions to two may indicate a lower training volume. Conversely, for another team, reducing from two sessions to one could represent a similar strategy for decreasing volume. Although both approaches result in a lower volume, they are not directly comparable; the lower volume in the first example is higher than the volume in the second. Nonetheless, in both cases, context determines the implementation of lower-volume training, which is essential for adapting training strategies to the specific needs of each team.

Lower-volume training is poised to offer a strategic avenue for program design, particularly when the overarching objective is to attain an effective training stimulus conducive to physiological and physical adaptations, all the while minimizing the interference effects and fatigue [13, 14]. However, the significance of lower-volume training goes beyond merely addressing the challenges posed by competitive schedules and program strategizing. One of the initial challenges is defining the primary objective of low-volume training. It is unclear whether it aims to maintain performance or to facilitate improvements, even if they are minimal. This fundamental question must be addressed when setting up a low-volume training program.

Furthermore, lower-volume training can seamlessly integrate with personalized strategies tailored to the unique requirements of each player. This entails inducing training doses that allow players to complement their in-field training regimen (i.e., practice sessions on the playing field focusing on tactical and technical drills), while adhering to the criteria of minimal individualized dosing, thereby ensuring the conditions for targeted adaptations are met [15]. This approach encourages players’ motivation by giving them the autonomy to choose longer or shorter training sessions based on their preferences. However, it poses a challenge for researchers as it makes it more difficult to control and ensure consistent exposure to training, thereby affecting the replicability of conditions.

This paradigm is exceptionally well-suited for out-field training contexts, particularly in scenarios in which players engage in customized training sessions under the guidance of personal trainers [16]. Moreover, lower-volume training is promising in individual settings, facilitating the administration of tailored doses that align with the player’s readiness and training status [17]. For example, specific lower doses can be introduced during periods of lower or higher fatigue, with the distribution being modulated accordingly.

Lower-volume training’s versatility is evidenced by its applicability across a wide spectrum of training types and modalities. The prominence of this concept is reflected in the increasing number of publications delving into its intricacies. For instance, studies have compared lower-volume training against regular or higher-volume training within contexts such as high-intensity interval training [18], plyometric training [5], strength training [4, 6], and sprint [19]. These studies have aimed to determine the effects of lower-volume training on crucial physical fitness parameters, including cardiorespiratory endurance performance, neuromuscular strength, power, and running speed [20–23].

In the context of team sports, these physical attributes are pivotal determinants bolstering players’ overall performance [24]. Consequently, the domain of lower-volume training offers a fertile ground for elucidating its efficacy across diverse athletes and team sports. This affords a significant opportunity to delve into the nuanced efficacy of lower-volume training, thereby contributing insights into its potential to optimize athletes’ performance across varying contexts [25].

Despite the extensive body of research on this topic [12, 26], the accumulation of systematic reviews dedicated specifically to team sports players remains limited. Various reviews have explored the dichotomy between lower-volume and higher-volume training in non-athletic populations, focusing on training types such as high-intensity interval training [27, 28] and strength training [12, 26], or even individual sports like swimming [29]. For instance, a meta-analysis [30] found that low-volume high-intensity interval training at higher intensities significantly improved cardiorespiratory fitness, while increasing repetitions, high-intensity duration, or total session length did not enhance these benefits. However, the context of team sports brings unique considerations, including the challenges posed by regular and densely competitive schedules [2] and the need to address multiple and potentially concurrent fitness components [31], which are of significant relevance to those who work regularly with team sport athletes.

A systematic review and subsequent meta-analysis provide an avenue to consolidate primary evidence regarding the impact of lower-volume training on the physical fitness adaptations of team sports players. Additionally, they could offer an encompassing overview of the methodological approaches employed in lower-volume training strategies, thus serving as a valuable resource for practitioners. Furthermore, they could lay the groundwork for identifying promising avenues for future research within this domain.

With these considerations in mind—and given the need to uncover the potential utility and effectiveness of lower-volume training in the context of team sports—the objective of this systematic review with meta-analysis is twofold: (i) to summarize and synthesize the principal physiological and physical fitness outcomes resulting from lower-volume training interventions among team sports players, encompassing various training modalities, and (ii) to compare the effects of lower-volume training against higher-volume training while accounting for the specific types of training approaches employed.

## Methods

This systematic review and meta-analysis was conducted as per the Cochrane guidelines [32] and reported as per the Preferred Reporting Items for Systematic Reviews and Meta-Analyses (PRISMA 2020) guidelines [33] and reporting guidelines for sports sciences reviews [34].

### Protocol and Registration

The systematic review's protocol underwent preliminary submission and was subsequently published on the Open Science Framework on the 08th of September 2023. The protocol is accessible through the following web address: https://doi.org/10.17605/OSF.IO/67G8T, and it can also be located using the registration number, osf.io/7s3un.

### Eligibility Criteria

The inclusion criteria encompassed original research studies published within peer-reviewed journals, encompassing those labeled as "in press" or "ahead-of-print." No other classifications of studies were considered. Additionally, research conducted in all languages was considered eligible for inclusion, with no temporal restrictions imposed [35].

Furthermore, we adhered to the PICOS (Population, Intervention, Comparator, Outcomes, Study design) framework to define and establish the precise eligibility criteria, as detailed in Table 1.

**Table 1:** Eligibility criteria for the systematic review

	Inclusion criteria	Exclusion criteria
Population	The pool of eligible participants comprised both men and women team sports players who did not report any existing injuries or illnesses. These individuals were required to be actively engaged in team training regimens and possess a minimum competitive level corresponding to Participants Classification Framework tier 2 (trained/developmental level) [36] and regardless of age	We excluded team sports players who were disabled, injured, or in an unhealthy state from consideration. Furthermore, athletes from sports other than team sports were outside the scope of this review. Lastly, participants classified in tiers 0 or 1 of the Participants Classification Framework were also excluded
Intervention	The review will include studies that have compared lower-volume training strategies with higher-volume training. Only studies that directly compare both approaches within a single experiment (i.e., 2-arm, parallel) will be included. This means that part of the sample must be exposed to a lower training volume, while another part is subjected to the same training method but with a higher volume, achieved through more repetitions, longer duration, and/or greater training frequency. In our current stage, we utilized volume as a definition derived from the product of exercise intensity, time, and frequency [10]. However, our particular emphasis is on time/repetitions, such as the duration of the session, or number of repetitions/sets and frequency, which refers to the number training sessions per week. These studies must offer comprehensive descriptions within their articles, with the stipulation that these descriptions are not confined solely to training duration. The level of information provided pertaining to the lower-volume training interventions should be sufficiently detailed. At a minimum, this information should encompass variables such as training frequency, the composition of the training regimen (e.g., repetitions/sets, training time), or discernible reductions in training load, effectively distinguishing it from the comparator, which comprises a higher-training volume. These studies may, if feasible, make comparisons with traditional, regular and/or higher-volume training approaches. It is important to note that this inclusion criterion remains independent of the specific training method utilized, whether it involves high-intensity interval training, strength and power training, sprint training, or any other training type. The intervention periods must be at least 2 weeks long	The review will exclude studies that do not compare lower and higher training volumes within the same study (e.g., studies comparing low training doses with a passive control group will be excluded). Furthermore, studies failing to offer unequivocal depictions of the lower-volume training approach or neglecting to substantiate discernible discrepancies in contrast to higher- or standard-volume training will also be excluded. For instance, studies lacking clarification on the variables—such as training frequency, regimen composition, training duration, or training load—where disparities exist between lower- and higher-volume training, will be subject to exclusion
Comparator	The eligibility criteria encompass regular training volume, high training volume, or any other training volume that exceeds the experimental lower-volume training. This criterion has been established to ensure an unbiased and equitable assessment of the two training modalities, thus promoting methodological consistency for a credible comparative analysis	Volume-equated training (e.g., 1 vs 2 training sessions, with the total training volume remaining constant). Moreover, the exclusion criteria concerning comparators encompassed studies that established an unequal juxtaposition between lower- and higher-volume or standard training regimens. This involved the utilization of disparate training methodologies not congruent with those employed within the lower-volume training group (for instance, resistance training versus high-intensity interval training). Finally, this review will exclude studies that conflate training volume variance with concurrent intensity effects (e.g., comparing high-intensity interval training at 90% of HRmax with continuous training at 70%, or strength training with resistance load vs. unloaded exercises)
Outcomes	The primary outcomes involve adaptations (medium to long term adaptations), concentrating on assessments of physiological or physical fitness levels at a minimum of two time points (baseline and post-intervention). These adaptations encompass various aspects, including cardiorespiratory endurance fitness, neuromuscular strength and power, running speed, change-of-direction ability, as well as flexibility and mobility levels or balance	The review will exclude studies that do not present crucial data such as mean values, standard deviations, or equivalent statistical metrics. Additionally, studies that did not conduct baseline or post-intervention analyses will also be excluded from consideration. This review will exclude acute-based physiological or physical parameters that do not contribute to a comprehensive understanding of physical fitness adaptations but instead reflect immediate training-induced changes in the player's response. Additional variables such as body composition, technical or tactical elements, and psycho-sociological factors will also be subjected to exclusion
Study design	Randomized or non-randomized with ≥ 2 arms. It is also possible to include crossover designs with washout periods of at least 2 weeks	Single-arm studies. Crossover designs without a washout period

PCF: participant classification framework; The competitive level was classified based on the Participant Classification Framework [36]: Tier 0 and Tier 1: sedentary and recreationally active (not included, considering the context of this systematic review); Tier 2: trained/developmental; Tier 3: highly trained/national level; Tier 4: elite/international level; Tier 5: world class

### Information Sources

The quest for pertinent studies involved searches across the following databases: PubMed, Scopus, SPORTDiscus, and Web of Science (Core Collection). These searches were executed on September 08, 2023, following the completion of the protocol registration (ID: osf.io/7s3un). No restrictions on publication dates were applied. Additionally, manual examinations of reference lists within included studies were conducted to identify potentially relevant titles. Subsequently, the abstracts of these articles were assessed against the relevant inclusion criteria, with full-text retrieval as needed. Furthermore, snowballing citation tracking was conducted, with a preference for utilizing Web of Science. To enhance the rigor of the review, insights were also sought from two external experts with global recognition, as verified by Expertscape (https://expertscape.com/ex/team+sports). As part of the review process, articles included in the review underwent scrutiny for potential errata or retractions [44].

### Search Strategy

The search process incorporated the application of Boolean operators AND/OR, with a deliberate decision to refrain from using filters or restrictions pertaining to date, language, or study design. This approach was adopted to maximize the potential for uncovering pertinent studies. The search strategy employed, serving as the principal means of identifying relevant studies, is as follows:

[Title/Abstract] “team sport*” OR football* OR soccer OR futsal OR handball* OR volleyball* OR basketball* OR hockey OR rugby OR cricket OR “water polo” OR lacrosse OR softball OR korfball

#### AND

[All fields/Full text] “low-volume” OR “low volume” OR “low training volume” OR “low training” OR “high versus low volume” OR “high versus low training volume” OR “training volume*” OR “lower frequency” OR “higher frequency” OR “low-frequency” OR “high-frequency” OR “low frequency” OR “high frequency” OR “micro-dos*” OR “micro dos*” OR “microdos*” OR “microtraining*” OR “microload*” OR “minimum dos*” OR “minimal dos*” OR “micro-priming” OR “minimal effective dos*” OR “minimum effective dos*” OR “minimum training dos*” OR “minimal effective dos*” OR “minimal training dos*” OR “minimum training dos*”.

The full search strategy can be observed in Table 2.

**Table 2:** Full search strategy for each database

Database	Specificities of the databases	Search strategy	Titles retrieved (n)
PubMed	None to report	("team sport*"[Title/Abstract] OR football*[Title/Abstract] OR soccer[Title/Abstract] OR futsal[Title/Abstract] OR handball*[Title/Abstract] OR volleyball*[Title/Abstract] OR basketball*[Title/Abstract] OR hockey[Title/Abstract] OR rugby[Title/Abstract] OR cricket[Title/Abstract] OR "water polo"[Title/Abstract] OR lacrosse[Title/Abstract] OR softball[Title/Abstract] OR korfball[Title/Abstract]) AND ("low-volume" OR "low volume" OR "low training volume" OR "low training" OR "high versus low volume" OR "high versus low training volume" OR "training volume*" OR "lower frequency" OR "higher frequency" OR "low-frequency" OR "high-frequency" OR "low frequency" OR "high frequency" OR "micro-dos*" OR "micro dos*" OR "microdos*" OR "microtraining*" OR "microload*" OR "minimum dos*" OR "minimal dos*" OR "micro-priming" OR "minimal effective dos*" OR "minimum effective dos*" OR "minimum training dos*" OR "minimal effective dos*" OR "minimal training dos*" OR "minimum training dos*")	550
Scopus	Search for title and abstract also includes keywords	( TITLE-ABS-KEY ( "team sport*" OR football* OR soccer OR futsal OR handball* OR volleyball* OR basketball* OR hockey OR rugby OR cricket OR "water polo" OR lacrosse OR softball OR korfball) AND ALL ("low-volume" OR "low volume" OR "low training volume" OR "low training" OR "high versus low volume" OR "high versus low training volume" OR "training volume*" OR "lower frequency" OR "higher frequency" OR "low-frequency" OR "high-frequency" OR "low frequency" OR "high frequency" OR "micro-dos*" OR "micro dos*" OR "microdos*" OR "microtraining*" OR "microload*" OR "minimum dos*" OR "minimal dos*" OR "micro-priming" OR "minimal effective dos*" OR "minimum effective dos*" OR "minimum training dos*" OR "minimal effective dos*" OR "minimal training dos*" OR "minimum training dos*"))	2743
SPORTDiscus	Duplicate search, breaking down for titles and then for abstracts, regarding the first line	TI (“team sport*” OR football* OR soccer OR futsal OR handball* OR volleyball* OR basketball* OR hockey OR rugby OR cricket OR “water polo” OR lacrosse OR softball OR korfball) AND TX (“low-volume” OR “low volume” OR “low training volume” OR “low training” OR “high versus low volume” OR “high versus low training volume” OR “training volume*” OR “lower frequency” OR “higher frequency” OR “low-frequency” OR “high-frequency” OR “low frequency” OR “high frequency” OR “micro-dos*” OR “micro dos*” OR “microdos*” OR “microtraining*” OR “microload*” OR “minimum dos*” OR “minimal dos*” OR “micro-priming” OR “minimal effective dos*” OR “minimum effective dos*” OR “minimum training dos*” OR “minimal effective dos*” OR “minimal training dos*” OR “minimum training dos*”) AND AB ( “team sport*” OR football* OR soccer OR futsal OR handball* OR volleyball* OR basketball* OR hockey OR rugby OR cricket OR “water polo” OR lacrosse OR softball OR korfball) AND TX (“low-volume” OR “low volume” OR “low training volume” OR “low training” OR “high versus low volume” OR “high versus low training volume” OR “training volume*” OR “lower frequency” OR “higher frequency” OR “low-frequency” OR “high-frequency” OR “low frequency” OR “high frequency” OR “micro-dos*” OR “micro dos*” OR “microdos*” OR “microtraining*” OR “microload*” OR “minimum dos*” OR “minimal dos*” OR “micro-priming” OR “minimal effective dos*” OR “minimum effective dos*” OR “minimum training dos*” OR “minimal effective dos*” OR “minimal training dos*” OR “minimum training dos*”)	2058 + 2591
Web of Science	Search for title and abstract also includes keywords and its designated “topic”	“team sport*” OR football* OR soccer OR futsal OR handball* OR volleyball* OR basketball* OR hockey OR rugby OR cricket OR “water polo” OR lacrosse OR softball OR korfball (Topic) and “low-volume” OR “low volume” OR “low training volume” OR “low training” OR “high versus low volume” OR “high versus low training volume” OR “training volume*” OR “lower frequency” OR “higher frequency” OR “low-frequency” OR “high-frequency” OR “low frequency” OR “high frequency” OR “micro-dos*” OR “micro dos*” OR “microdos*” OR “microtraining*” OR “microload*” OR “minimum dos*” OR “minimal dos*” OR “micro-priming” OR “minimal effective dos*” OR “minimum effective dos*” OR “minimum training dos*” OR “minimal effective dos*” OR “minimal training dos*” OR “minimum training dos*” (All Fields)	931

### Selection Process

The records obtained, encompassing titles and abstracts, underwent an independent screening process conducted by two authors (FMC and RMS). These same authors also individually reviewed the full texts of the selected studies. In instances where disparities emerged, the two authors engaged in discussions and re-evaluation of the studies collaboratively. When a consensus remained elusive, a third author (PZ) was consulted to render the final verdict. Throughout this selection phase, all co-authors contributed their perspectives and assistance as required. For efficient management and deduplication of records, both manual and automated procedures were employed, facilitated by EndNote™ 20.5 software from Clarivate™.

### Data Collection Process

The lead author (FMC) initiated the initial data extraction process, which subsequently underwent a review for both accuracy and comprehensiveness by two co-authors (RMS and PZ). To facilitate this task, a dedicated Microsoft® Excel datasheet was designed, encompassing all pertinent data and essential information. A representative sample of this Excel datasheet is included in the Supplementary Material 1. In scenarios where vital data were absent from the full text, the primary author (FMC) took proactive steps by directly contacting the corresponding author of the study, employing means such as email and ResearchGate, to solicit the required information. If the authors did not respond, the article was excluded from the systematic review integration. This did not occur.

### Data Items

To provide a comprehensive contextual overview, the compilation of data pertaining to studies and participants encompassed the following variables: the sport discipline, age, sex, competitive level as delineated by the Participant Classification Framework (PCF) [36], standard training frequency, and volume within their respective club environments. The classification of training volume into "lower" and "higher" was determined based on the individual studies. In each study, the intervention with fewer repetitions, shorter duration, and/or lower training frequency was classified as the lower training volume. Conversely, the intervention with more repetitions, longer duration, and/or a higher frequency of weekly sessions was categorized as the higher training volume. This classification was applied within each study, meaning it was specific to the study’s context and not generalized across multiple studies as a single, uniform term or dose.

It is worth mentioning that training volume can be contingent upon the type of training, as demonstrated by examples such as endurance-based training, where the outcome might be influenced by the duration of work undertaken. Conversely, in resistance training, it could be tied to the cumulative count of repetitions and sets executed. Similarly, in the context of sprint-running training, the outcome might be associated with the total distance covered during sprints. It is noteworthy that these data elements remain distinct from the intervention-specific details. Furthermore, the temporal aspects of the season, encompassing phases such as the competitive season and the off-season, were integrated as crucial components of the contextual framework. This inclusion contributed to a more comprehensive grasp of the study's findings.

Participant randomization was duly registered as a component of the study protocol. The competitive level was classified based on the Participant Classification Framework [36]: Tier 0 and Tier 1: sedentary and recreationally active (not included, considering the context of this systematic review); Tier 2: trained/developmental; Tier 3: highly trained/national level; Tier 4: elite/international level; Tier 5: world class. Additionally, any potential competing interests and details regarding funding sources were thoroughly documented and disclosed.

Intervention-related information: The documentation pertaining to the training intervention encompassed an extensive array of variables. These variables encompassed, although were not constrained to, adherence and compliance rates, the specific type and modality of training employed, the program's duration measured in weeks, the aggregate count of training sessions, the frequency of these sessions (sessions per week), and the training duration (illustratively measured in minutes per session), or training volume (illustratively measured in repetitions, sets or time of work, or distance covered, tailored to the specificities of cardiorespiratory, resistance-based, or sprint training, respectively).

Additionally, the precise training prescription was recorded, detailing factors such as sets, repetitions per set, the duration of each repetition, and recovery periods both between and within sets. The intensities of training were documented, as well as the type of field or surface utilized for the training sessions, whether synthetic or natural turf. Other parameters, such as training load (if measured), were also collated.

In the context of comparing lower and higher-volume training groups, efforts were made to elucidate the degree of increased volume present within the higher-volume group when juxtaposed against the lower-volume training intervention group, should these data be available.

Outcomes: The primary outcomes pertain to adaptations, centering on evaluations of physiological and/or physical fitness levels conducted at a minimum of two time points (baseline and post-intervention). These adaptations encompass a range of aspects, including cardiorespiratory endurance fitness (measured by direct or indirect measures, as field-based tests), neuromuscular strength and power, running speed, change-of-direction ability, as well as flexibility and mobility levels and balance.

Outcome-related information: to ensure a comprehensive grasp of the outcomes, the information related to outcomes will encompass several crucial components. These include contextual details surrounding the assessment, such as the duration of rest preceding the analysis and the precise time of day during which the testing was conducted. Furthermore, the inclusion or exclusion of a familiarization period prior to the physical tests will be documented, as it has the potential to impact participant performance. Additionally, meticulous consideration will be given to the implementation of blinding procedures. These procedures are crucial to maintain observer impartiality, ensuring that those conducting the tests are uninfluenced by any prior knowledge of the test conditions.

### Study Risk of Bias Assessment

Employing Cochrane's Risk of Bias tool, version 2 (RoB 2) [37], we conducted assessments on parallel randomized studies, considering bias in five distinct domains: randomization process, adherence to intended interventions (including intention-to-treat analysis), handling of missing outcome data, outcome measurement, and selection of reported results. For non-randomized studies, we employed Cochrane's Risk of Bias In Non-Randomized Studies of Interventions (ROBINS-I) [38], assessing bias across seven domains: confounding, participant selection, intervention categorization, adherence to intended interventions, handling of missing data, outcome measurement, and selection of reported results.

Our evaluation of bias was conducted both at the outcome level and the study level, presenting the most adverse case scenario per individual study. In the absence of a pre-registered protocol, we categorized the risk of bias related to the selection of reported results as at least having some concerns (RoB 2) or presenting a moderate risk (ROBINS-I). To ensure rigor, two authors (FMC and RMS) conducted independent assessments of bias, with a third author (PZ) acting as an arbitrator when necessary. A comprehensive summary of risk of bias evaluations was subsequently provided, organized according to the main outcome measures.

### Summary Measures, Synthesis of Results, and Publication Bias

We performed meta-analyses only when at least three studies were available [39] per each physical fitness component in accordance with the Cochrane Handbook [40]. Hedges' *g* effect sizes (ES), with 95% confidence interval (CI) and 95% prediction interval (PI), were computed for the physical fitness variables within both the lower-volume training intervention and comparator groups. These ES values were determined using the means and standard deviations derived from pre- and post-intervention measurements. The data were standardized using the post-intervention standard deviation values. To address any inherent disparities across studies that might influence the small-study effects (SSE) impact, we applied the DerSimonian and Laird random-effects model. This statistical approach aids in accounting for variations between studies and supports the robustness of the overall findings [41, 42].

The ES values were presented with 95% confidence intervals (95% CIs), and their interpretation was based on the following scale: 0.0–0.2 trivial, 0.2–0.6 small, > 0.6–1.2 moderate, > 1.2–2.0 large, > 2.0–4.0 very large, > 4.0 extremely large [43]. For studies that included more than one intervention group, the sample size in the control group was proportionally divided to facilitate comparisons across multiple groups [45]. To assess the impact of heterogeneity, we used *I*^2^ statistics, with values of < 25%, 25–75%, and > 75% representing low, moderate, and high impact of heterogeneity, respectively [46].

We explored the risk of publication bias for continuous variables (≥ 10 studies per outcome) using the extended Egger's test [47], and to adjust for this risk, we conducted a sensitivity analysis using the trim and fill method [48] with L0 as the default estimator for the number of missing studies [49]. All analyses were conducted using the Comprehensive Meta-Analysis Software (Version 4, Biostat, Englewood, NJ, USA), and statistical significance was set at *p* ≤ 0.05.

### Subgroup Analyses

In this study, we identified potential sources of heterogeneity that were likely to exert influence on the effects of the training interventions. Acknowledging that adaptive responses to intervention programs can be modulated by individual factors such as training type, team sport involvement, sex, competitive level [50], and the total number of sessions administered (or total volume of training), we systematically considered these as prospective moderator variables. Additionally, the primary physical fitness outcome type was also accounted for in the partitioned analysis.

Regarding the amalgamation of outcomes, it is imperative to highlight that the analysis was conducted with respect to the specific training types elucidated in the studies. This approach entailed the aggregation of all resistance training studies, alongside the grouping of studies focused on low-volume endurance training, low-volume speed training, and other pertinent categories.

### Single Training Factor Analyses

In performing subgroup analyses and when delving into single training factor investigations, we employed the median split technique [51–53] as deemed appropriate. To execute this technique, it was essential that a minimum of three studies furnished pertinent data for a particular moderator variable. This approach was adopted to avoid any undue inflation of the median calculation's impact.

Furthermore, when deriving median values, we refrained from employing a universal median value sourced from all encompassed studies (e.g., median age derived from all studies under consideration). Instead, we meticulously calculated median values, exclusively incorporating studies that provided data pertinent to the specific outcome being analyzed.

In instances where the application of the median split technique was deemed unsuitable, we exercised discernment in determining the rationale for conducting subgroup analyses, ensuring a sound and reasoned approach.

### Sensitivity Analyses

Sensitivity analyses were systematically undertaken to ascertain the resilience of the summary estimates, encompassing metrics like p-values, effect sizes, and the I^2^ statistic. In a bid to gauge the influence of individual studies on the overarching conclusions, we executed an automated leave-one-out analysis. Within this analysis, each study's outcomes were examined iteratively with that particular study omitted from the model.

This meticulous process granted us the ability to gauge the distinct impact of each individual study on the summary estimates. Furthermore, it afforded a comprehensive assessment of the overall robustness of our findings, thus enhancing the confidence in the reliability of our conclusions.

### Certainty Assessment

Employing the Grading of Recommendations Assessment, Development and Evaluation methodology (GRADE) [54], two authors (FMC and HS) undertook the evaluation of evidence certainty while effectively resolving any differences through consensus. This comprehensive assessment concentrated on four of the five dimensions integral to the GRADE framework [55, 56]: risk of bias, inconsistency, potential for publication bias, and imprecision.

Based on the comprehensive evaluation of these four domains, the GRADE framework assigns a quality rating—ranging from high to very low—to the body of evidence concerning each outcome of interest. This rating system serves as a navigational tool, guiding both recommendations for practical application and directions for future research endeavors.

In the context of non-randomized studies, the evaluation initially began with a very low level of evidence. However, these evaluations could be upgraded considering several factors. These factors encompassed the identification of significant effect sizes, adept control over potential confounding variables, and the substantiation of a dose–response gradient. This process led to elevations in the evidence quality rating from its initial low level in the case of non-randomized studies.

## Results

### Study Selection

The initial database search resulted in 8873 records, and upon review, 3687 of these were identified as duplicates. Following a thorough screening of the remaining 5186 records, applying specific criteria such as article type or PICOS, we excluded 5000 records. This screening process was conducted from September 29, 2023, to October 03, 2023.

Subsequently, we performed a comprehensive analysis of the full text for 186 studies. Among these, 16 studies met the predefined eligibility criteria and were consequently incorporated into the review. The remaining 170 studies were excluded for various reasons, which can be referenced in supplementary material 1. This phase of full-text analysis extended from October 04, 2023, to October 19, 2023.

**Fig. 1:**
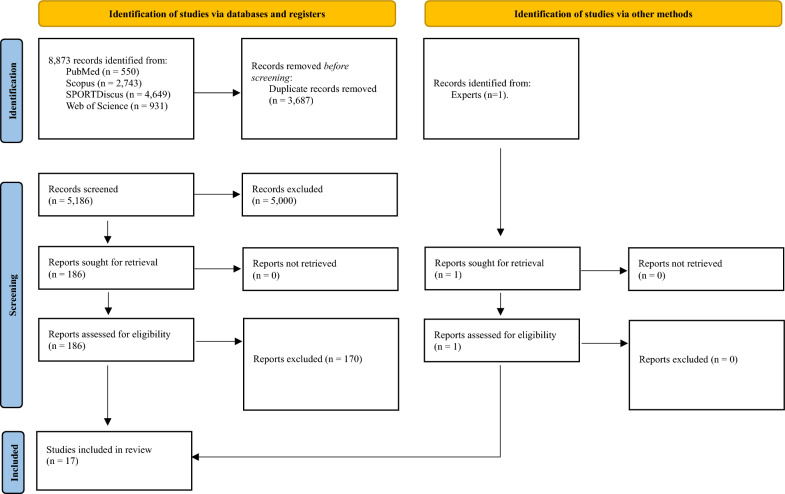
PRISMA 2020 flow diagram

Additionally, it is noteworthy that two independent researchers, recognized experts in the field, identified an additional eligible article, which was subsequently confirmed through thorough full-text analysis. Consequently, the final compilation for this systematic review consists of a total of 17 articles, as depicted in Fig. 1.

### Study Characteristics

Table 3 offers a comprehensive summary of the primary study design characteristics found in the studies included in this systematic review. Of the included studies, 12 were centered on soccer players. In terms of the competitive level, three studies examined players in tier 3 (highly trained/national level), whereas the remaining 14 studies focused on tier 2 players (trained/developmental). The number of participants per study ranged from a minimum of 18 [57] to a maximum of 158 [58]. Regarding sex representation, 10 studies were exclusively centered on men, with one exclusively focused on women, and one study integrated both men and women in the same experiment. The remaining five studies did not report the sex of the sample.

**Table 3:** Descriptive characteristics of study designs, participants, interventions, and outcomes of the included articles

Study	Sport	Competitive level	N	Age (years old)	Sex	Type of study	Randomization	Compliance rates (%)	Season period	Tests	Outcomes extracted
Beltran-Valls et al. [60]	Soccer	Tier 2	22	23.0 ± 5.0	Men	Two-arm	Yes	ND	ND	CMJ test; Illinois COD test; 10-m sprint test	CMJ power (W/kg); COD time (s); 10-m sprint time (s)
Bianchi et al. [5]	Soccer	Tier 2	21	17.0 ± 1.0	ND	Two-arm	Yes	ND	Pre-season	Long jump test; Single leg triple hop test; 10-, 30- and 40-m sprint test; 5-0-5 COD test	Horizontal jump (cm); Triple hop right and left (m); 10-, 30- and 40-m sprint time (s); 5-0-5 COD time (s)
Chaabene et al. [71]	Soccer	Tier 2	25	12.7–12.7	Men	Two-arm	Yes	At least 80%	2nd half of the season	CMJ Test; SJ test; Standing long jump; T-test; 5-, 10-, 20- and 30-m sprint test	CMJ jump height (cm); SJ height (cm) Horizontal jump (m); T-test COD time (s); 5-, 10-, 20- and 30-m sprint time (s)
Coutts et al. [57]	Rugby	Tier 2	18	23.3 ± 3.3	Men	Two-arm	Yes	ND	ND	All-out cycle sprint; Vertical jump test; Incremental treadmill test	Peak cycling power (W); Vertical jump height (cm); VO2max (ml/kg/min)
Fortes et al. [61]	Futsal	Tier 2	78	12–17	Men	Two-arm	Yes	At least 95%	ND	YYIRT level 1	YYIRT distance (m)
Hoffman et al. [59]	American Football	Tier 2	61	19.7–20.1	ND	Four-arm	No	ND	Winter season	Bench press 1RM; Squat 1RM; 40-yard sprint test; Vertical jump test; 2-mile run test	Bench press 1RM (kg); Squat 1RM (kg); 40-yard sprint time (s); Vertical jump height (cm); 2-mile test time (s)
Krespi et al. [58]	Soccer	Tier 2	158	17.1 ± 0.8	Men	Two-arm	Yes	ND	ND	5-, 10-, 30-m sprint test; Sprint “96,369” with 180º turn; SJ test; CMJ test; Incremental treadmill test; RSA test	5-, 10-, 20- and 30-m sprint time (s); COD time (s); SJ height (cm); CMJ height (cm); VO2max (ml/kg/min); RSA (s)
Lacome et al. [67]	Soccer	Tier 2	19	17.2–17.5	ND	Two-arm	Yes	ND	ND	Knee-flexor eccentric strength	Eccentric strength (N)
Naclerio et al. [68]	Soccer and Volleyball	Tier 2	32	23.3–23.9	Men and women	Four-arm	Yes	ND	ND	Bench press 1RM; Upright row 1RM; Squat 1RM	Bench press 1RM (kg); Upright row 1RM (kg); Squat 1RM (kg)
Otero-Esquina et al. [70]	Soccer	Tier 2	36	17.0 ± 1.0	Men	Three-arm	No	ND	ND	CMJ test; 10- and 20-m sprint test; V-cut COD test	CMJ power (W/kg); 10- and 20-m sprint time (s); COD time (s)
Palma-Muñoz et al. [65]	Basketball	Tier 2	22	13.5 ± 2.0	Men	Three-arm	Yes	ND	ND	CMJ test; Horizontal bilateral test; 10-m sprint test; T-test	CMJ height (cm) Horizontal jump (cm) 10-m sprint time (s) COD time (s)
Ramírez-Campillo et al. [63]	Soccer	Tier 2	23	21.4 ± 3.2	Women	Three-arm	Yes	At least 87%	ND	CMJ test; 15-m sprint test; Meylan COD test; YYIRT level 1	CMJ height (cm); 15-m sprint time (s); COD time (s); YYIRT distance (m)
Ramírez-Campillo et al. [64]	Soccer	Tier 2	24	13.0 ± 2.3	Men	Three-arm	Yes	ND	ND	CMJ free arms; Horizontal bilateral jump free arms; 10-m sprint test; T-test; YYIRT level 1	CMJ free arms height (cm); Horizontal jump free arms (cm); 10-m sprint time (s); COD time (s); YYIRT distance (m)
Rebaï et al. [62]	Soccer	Tier 2	20	18.4 ± 0.8	Men	Two-arm	Yes	ND	Ramadan period	SJ test; CMJ test; Maximal voluntary contraction of the knee extensors	SJ height (cm); CMJ height (cm); Knee extensors strength (N)
Severo-Silveira et al. [69]	Rugby	Tier 3	21	19–33	ND	Two-arm	Yes	ND	ND	Knee flexor/extensor strength	Knee extensor concentric peak torque (Nm); Knee flexor concentric peak torque (Nm); Knee flexor eccentric peak torque (Nm)
Shi et al. [18]	Soccer and handball	Tier 2	32	19.5–20.6	Men	Three-arm	Yes	ND	Off-season and pre-season	YYIRT level 1 RSA	YYIRT distance (m) Maximal sprint velocity at RSA (m/s) VO2max (ml/kg/min)
Yanci et al. [66]	Soccer	Tier 3	16	22.5–24.6	Men	Two-arm	Yes	ND	In-season	5-, and 15-m sprint test; Modified agility test free; Horizontal jump test; CMJ test; YYIRT level 1	5- and 15-m sprint time (s); COD time (s); Horizontal jump (cm); CMJ flight time (s); YYIRT distance (m)

CMJ, countermovement jump; COD, change-of-direction; ND, not described; RM, repetition maximum; RSA, repeated sprint ability; SJ, squat jump; VO2max, maximal oxygen uptake; YYIRT, Yo-Yo Intermittent recovery test

The most prevalent study design was a two-arm study (n = 10), followed by a three-arm design (n = 5). In terms of randomization, only one study [59] did not explicitly declare randomization, while the remainder (n = 16) reported using randomization to assign players to the groups. Out of the studies providing information about the context of experimental implementation within the sports season, only 6 reported the phase of the season.

Table 4 provides a summary of the methodological characteristics of the training programs in the included studies. Among these studies, four implemented interventions during tapering periods occurring after equal-volume training interventions [58, 60–62], and three studies compared constant versus progressive load training [63–65]. The remaining studies focused on interventions without a periodized approach, such as tapering or progressive load.

**Table 4:** Description of the characteristics of the training programs in the individual studies included in the systematic review

Study	Group classification	Participants (n)	Context	Training method	Duration (w)	Frequency (s/w)	Sets per week	Repetitions per week	Time per week	Difference between lower and higher volume
Beltran-Valls et al. [60]	LV	11	Tapering after 6-week training	Plyometrics, COD and SSGs	2	4	ND	ND	ND	− 2.1 × in total time of session Training load: 344–372 AU/week
Beltran-Valls et al. [60]	HV	11	Tapering after 6-week training	Plyometrics, COD and SSGs	2	4	ND	ND	ND	+ 2.1 × in total time of session Training load: 746.2–749.7 AU/week
Bianchi et al. [5]	LV	10	New intervention	Plyometric training	8	1	8	44	20	− 2 × in total number of repetitions
Bianchi et al. [5]	HV	11	New intervention	Plyometric training	8	2	16	88	40	+ 2 × in total number of repetitions
Chaabene et al. [71]	LV	13	New intervention	Plyometric training	8	2	5–8	100–240 week	ND	− 1.8 × of the total number of foot contacts (50–120)
Chaabene et al. [71]	HV	12	New intervention	Plyometric training	8	2	8–14	216–420 week	ND	+ 1.8 × of the total number of foot contacts (110–220)
Coutts et al. [57]	LV	9	New intervention	Field sessions and resistance training	6	5–7	ND	ND	ND	− 1.2 × of training load (11,445 AU) than the HV training group
Coutts et al. [57]	HV	9	New intervention	Field sessions and resistance training	6	5–7	ND	ND	ND	+ 1.2 × of training load (13,596) than the LV training group
Fortes et al. [61]	LV	47	Tapering after 8-week training	Physical, technical and tactical training	3	5	49	ND	ND	− 20 to 60% of less training volume than HV training group
Fortes et al. [61]	HV	47	Tapering after 8-week training	Physical, technical and tactical training	3	5	65	ND	ND	+ 20 to 60% of training volume than LV training group
Hoffman et al. [59]	MV	12	New intervention	Resistance training program	10	3	150–162	1,404–1,380	ND	− 1.1 × of total number repetitions than HV group
Hoffman et al. [59]	LV	15	New intervention	Resistance training program	10	4	118–126	1,116–1,000	ND	− 1.3 to 1.5 × of the total number of repetitions than HV group
Hoffman et al. [59]	MV	23	New intervention	Resistance training program	10	5	148–156	1,400–1,280	ND	− 1.1 to 1.2 × of total number repetitions than HV group
Hoffman et al. [59]	HV	11	New intervention	Resistance training program	10	6	154–162	1,476–1,460	ND	+ 1.3 to 1.5 × of the total number of repetitions than LV group
Krespi et al. [58]	LV	79	Tapering after 4-week training	Running exercises (90–95%HRmax)	4	3	3–12	ND	4–16	− 1.25 × of the total work time over the 4 weeks (96 min) than HV training group
Krespi et al. [58]	HV	79	Tapering after 4-week training	Running exercises (90–95%HRmax)	4	3	3–12	ND	4–16	+ 1.25 × of the total work time over the 4 weeks (120 min) than LV training group
Lacome et al. [67]	LV	9	New intervention	Eccentric training	6	1	2	10	ND	− 4 × less repetitions week in comparison to HV training
Lacome et al. [67]	HV	10	New intervention	Eccentric training	6	1	8	40	ND	+ 4 × of the total repetitions week than LV training group
Naclerio et al. [68]	LV	6	New intervention	Resistance training program (75%1RM)	6	3	27	216	ND	− 3 × less than the HV training group
Naclerio et al. [68]	MV	6	New intervention	Resistance training program (75%1RM)	6	3	54	432	ND	− 1.5 × less than the HV training group
Naclerio et al. [68]	HV	8	New intervention	Resistance training program (75%1RM)	6	3	81	648	ND	+ 3 × of total repetitions than LV training group
Otero-Esquina et al. [70]	LV	12	New intervention	Compound training, which encompasses strength training, plyometrics, and resisted sprinting	7	1	69	242	ND	− 2 × less than the HV training group in comparison to number of repetitions per week
Otero-Esquina et al. [70]	HV	12	New intervention	Compound training, which encompasses strength training, plyometrics, and resisted sprinting	7	2	138	484	ND	+ 2 × of total repetitions than LV training group in comparison to number of repetitions per week
Palma-Muñoz et al. [65]	LV	8	New intervention (constant load)	Plyometric training	6	2	48	240	ND	− 1.5 × of total repetitions during the training intervention (1,440 repetitions) in comparison to HV training group
Palma-Muñoz et al. [65]	HV	7	New intervention (progressive load)	Plyometric training	6	2	48	240–336	ND	+ 1.5 × of total repetitions during the training intervention (2,160 repetitions) in comparison to LV training group
Ramírez-Campillo et al. [63]	LV	8	New intervention	Plyometric training	8	1	5	40–70	ND	− 2 × of total repetitions during the training intervention (405 repetitions) in comparison to HV training group
Ramírez-Campillo et al. [63]	HV	8	New intervention	Plyometric training	8	2	10	80–140	ND	+ 2 × of total repetitions during the training intervention (810 repetitions) in comparison to HV training group
Ramírez-Campillo et al. [64]	LV	8	New intervention (constant load)	Plyometric training	6	2	48	240	ND	− 1.5 × of total repetitions during the training intervention (1,440 repetitions) in comparison to HV training group
Ramírez-Campillo et al. [64]	HV	8	New intervention (progressive load)	Plyometric training	6	2	48	240–480	ND	+ 1.5 × of total repetitions during the training intervention (2,160 repetitions) in comparison to LV training group
Rebaï et al. [62]	LV	10	Tapering after 4-week training	Resistance training program	4	3	9	8 reps/exercise/session	ND	− 1.9 × less training volume (5230.61 kg/week) in comparison to HV training group)
Rebaï et al. [62]	HV	10	Tapering after 4-week training	Resistance training program	4	3	12	8 reps/exercise/session	ND	+ 1.9 × more training volume (9893.75 kg/week) in comparison to LV training group)
Severo-Silveira et al. [69]	LV	10	New intervention (constant load)	Eccentric training	8	2	4–6	24–36	ND	− 1.8 × less total repetitions during the intervention (276 n) than the HV training group
Severo-Silveira et al. [69]	HV	11	New intervention (progressive load)	Eccentric training	8	2	4–8	24–80	ND	+ 1.8 × more total repetitions during the intervention (488 n) than the LV training group
Shi et al. [18]	LV	10	New intervention	Repeated sprint training in hypoxia	2	3	15	ND	75 s	− 2.5 × less sprint time during the intervention period (150 s) than HV training group
Shi et al. [18]	HV	10	New intervention	Repeated sprint training in hypoxia	5	3	15	ND	75 s	+ 2.5 × more sprint time during the intervention period (375 s) than LV training group
Yanci et al. [66]	LV	8	New intervention	Plyometric training	6	2	18	ND	ND	− 2.5 × foot contacts (180 n) than HV training group
Yanci et al. [66]	HV	8	New intervention	Plyometric training	6	2	36	ND	ND	+ 2.5 × foot contacts (360 n) than LV training group

Context: The context was categorized as follows: new intervention (for groups exposed to a new intervention), progressive intervention (when an increased volume was introduced), or tapering (in cases where the load decreased after a period of regular training); Group: The groups were classified into lower-volume (LV: indicating the group with a lower training volume), higher-volume (HV: representing the group with a higher training volume), and, in the case of a three-arm design, control group (if they received no intervention) or medium volume (MV: for groups with an intermediate volume); AU: arbitrary units; COD: change-of-direction; ND: not described; SSGs: small-sided games; s/w: session/week; W: weeks

In terms of training methods, five studies exclusively examined different volumes of plyometric training [5, 63–66], while five exclusively tested various volumes of resistance training (including concentric and eccentric emphasis) [59, 62, 67–69]. Running-based training methods were exclusively analyzed in two studies [18, 58].

The duration of the interventions often lasted 6 weeks, with the shortest period being 2 weeks [60] and the longest being 10 weeks [59]. Regarding training volume, the differences between lower-volume and higher-volume training groups ranged from 1.2 [57] to 4 times [67], with eight studies implementing less than a twofold difference between lower and higher-volume training.

### Risk of Bias in the Individual Studies

The risk of bias for the randomized studies was evaluated using the RoB2 instrument, and the findings are presented in Table 5. In the analysis of jump performance studies, 6 out of 10 exhibited an overall high risk of bias. Similarly, among studies examining change of direction performance, 5 out of 8 had an overall high risk of bias. For sprint performance studies, 4 out of 7 were found to have an overall high risk of bias. In the case of strength performance studies, 3 out of 4 demonstrated an overall high risk of bias. Among anaerobic power performance studies, 2 out of 3 had an overall high risk of bias, while only 1 out of 5 studies analyzing aerobic performance exhibited an overall high risk of bias. Finally, among studies analyzing VO2max performance, 1 out of 2 displayed an overall high risk of bias.

**Table 5:** Assessment of risk of bias for the randomized trials (RoB2)

Study	D1	D2	D3	D4	D5	Overall
Jumping performance						
Beltran-Valls et al. [60]	!	−	−	−	!	−
Bianchi et al. [5]	!	!	+	−	!	−
Chaabene et al. [71]	−	!	+	!	!	−
Coutts et al. [57]	−	!	+	−		−
Krespi et al. [58]	!	!	−	!	!	−
Palma-Muñoz et al. [65]	!	+	+	+	!	!
Ramírez-Campillo et al. [63]	+	+	+	+	!	!
Ramírez-Campillo et al. [64]	+	+	+	+	!	!
Rebaï et al. [62]	−	+	−	−	!	−
Yanci et al. [66]	!	+	+	!	!	!
Change-of-direction						
Beltran-Valls et al. [60]	!	−	−	−	!	−
Bianchi et al. [5]	!	!	+	−	!	−
Chaabene et al. [71]	−	!	+	!	!	−
Krespi et al. [58]	!	!	−	!	!	−
Palma-Muñoz et al. [65]	!	+	+	+	!	!
Ramírez-Campillo et al. [63]	+	+	+	+	!	!
Ramírez-Campillo et al. [64]	+	+	+	+	!	!
Yanci et al. [66]	!	+	+	−	!	−
Sprint performance						
Beltran-Valls et al. [60]	!	−	−	−	!	−
Bianchi et al. [5]	!	!	+	−	!	−
Chaabene et al. [71]	−	!	+	!	!	−
Krespi et al. [58]	!	!	−	!	!	−
Palma-Muñoz et al. [65]	!	+	+	+	!	!
Ramírez-Campillo et al. [63]	+	+	+	+	!	!
Ramírez-Campillo et al. [64]	+	+	+	+	!	!
Maximal Strength						
Lacome et al. [67]	−	+	+	!	!	−
Naclerio et al. [68]	−	−	−	+	!	−
Rebaï et al. [62]	−	+	−	−	!	−
Severo-Silveira et al. [69]	!	+	+	!	!	!
Anaerobic power						
Coutts et al. [57]	−	!	+	−	!	−
Krespi et al. [58]	!	!	−	!	!	−
Shi et al. [18]	+	+	!	!	!	!
Aerobic performance						
Fortes et al. [61]	−	!	−	−	!	−
Ramírez-Campillo et al. [63]	+	+	+	+	!	!
Ramírez-Campillo et al. [64]	+	+	+	+	!	!
Shi et al. [18]	+	+	!	!	!	!
Yanci et al. [66]	!	+	+	!	!	!
Coutts et al. [57]	−	!	+	−	!	−
Krespi et al. [58]	!	!	−	!	!	−

+, low risk; !, some concerns; −, high risk; D1, randomization process; D2, deviations from the intended interventions; D3, missing outcome data; D4, measurement of the outcome; D5, selection of the reported result

The high risk of bias was predominantly influenced by concerns in dimensions 1, 3, and 4, which pertain to insufficient information about randomization techniques and allocation concealment, missing data reports, and outcome measurement. Notably, the articles lacked adequate details on the random allocation of groups and effective concealment until the trials. Inconsistent concerns were observed in dimension 3, related to missing data reports, indicating a lack of information about data availability for all participants and potential bias in reported results due to missing outcome data. A consistent concern across studies was noted in dimension 4, regarding the blinding of assessors to tests and interventions. Many studies did not implement blinding measures, introducing the possibility of biased outcome assessments.

Dimension 5, which deals with the selection of reported results, also raised some concerns. The primary reason for these concerns was the absence of information about pre-specified analyses, making it unclear whether the reported results were selectively chosen from a larger set of outcomes. Overall, the risk of bias assessment suggests that the majority of included studies had limitations in crucial methodological aspects, particularly in randomization, allocation concealment, blinding, and result reporting.

The present systematic review incorporates an assessment of bias risk in non-randomized studies, employing Cochrane's Risk of Bias in Non-randomized Studies of Interventions (ROBINS-I) tool (Table 6). The results reveal that the non-randomized studies were categorized as having a serious overall risk of bias. This classification predominantly stemmed from the bias in the classification of intervention groups, and the lack of control of confounding variables.

**Table 6:** Assessment of risk of bias for non-randomized studies (ROBINS)

Study	Bias due to confounding	Bias in selection of participants into the study	Bias in classification of interventions	Bias due to deviations from intended interventions	Bias due to missing data	Bias in measurement of outcomes	Bias in selection of the reported result	Overall bias
Hoffman et al. [59]	Moderate	Low	Serious	Serious	Serious	Moderate	Low	Serious
Otero-Esquina et al. [70]	Moderate	Low	Serious	Low	Low	Moderate	Low	Serious

### Results of Individual Studies

Table 7 presents the results of individual studies conducted using resistance-based training. In both long jump and vertical jump performance, lower-volume and higher-volume training showed similar effects. Specifically, the improvements in long jump performance ranged from 6.3% [65] to 6.5% [5] for lower-volume training and from 5.5% [5] to 7.4% [65] for higher-volume training. For countermovement jump height, the enhancements ranged from 10.1% [70] to 13.5% [71] for lower-volume training and from 8.3% [70] to 14.1% [65] for higher-volume training.

**Table 7:** Results of individual studies—resistance-based training

Study	Context	Training type	Variable	LV (n)	LV pre	LV post	% (post–pre)	HV (n)	HV pre	HV post	% (post–pre)
*Horizontal jumps*											
Bianchi et al. [5]	New intervention	Plyometric training	Long jump (cm)	10	2.33 ± 0.15	2.48 ± 0.21	6.5⇑	11	2.18 ± 0.14	2.30 ± 0.17	5.5⇑
Bianchi et al. [5]	New intervention	Plyometric training	Triple hop right (m)	10	7.02 ± 0.72	7.25 ± 0.56	3.3⇑	11	6.81 ± 0.52	6.96 ± 0.59	2.2⇑
Bianchi et al. [5]	New intervention	Plyometric training	Triple hop left (m)	10	6.90 ± 0.60	7.18 ± 0.66	4.1⇑	11	6.75 ± 0.70	6.96 ± 0.68	3.1⇑
Chaabene et al. [71]	New intervention	Plyometric training	Standing long jump (m)	13	1.77 ± 0.09	1.89 ± 0.08	6.8⇑	12	1.64 ± 0.18	1.76 ± 0.19	7.3⇑
Palma-Muñoz et al. [65]	New intervention	Plyometric training	Horizontal jump, right leg (cm)	8	138.2 ± 35.2	144.1 ± 46.5	4.3⇑	7	137.1 ± 29.8	155.5 ± 30.5	13.4⇑
Palma-Muñoz et al. [65]	New intervention	Plyometric training	Horizontal jump, left leg (cm)	8	135.6 ± 42.8	154.2 ± 45.2	13.7⇑	7	141.9 ± 24.6	164.2 ± 28.1	15.6⇑
Palma-Muñoz et al. [65]	New intervention	Plyometric training	Horizontal jump (cm)	8	162.5 ± 42.5	172.7 ± 54.8	6.3⇑	7	161.1 ± 29.8	173.0 ± 36.1	7.4⇑
Ramírez-Campillo et al. [64]	New intervention	Plyometric training	Horizontal CMJ with arms (cm)	8	163 ± 42.6	170.5 ± 44.6	4.6⇑	8	160 ± 27.9	172.6 ± 30.1	7.9⇑
Ramírez-Campillo et al. [64]	New intervention	Plyometric training	Horizontal CMJ with arms, right leg (cm)	8	138 ± 35.3	141.9 ± 36.3	2.7⇑	8	138 ± 27.7	156.6 ± 31.4	13.6⇑
Ramírez-Campillo et al. [64]	New intervention	Plyometric training	Horizontal CMJ with arms, left leg (cm)	8	136 ± 42.9	155.2 ± 49.0	14.2⇑	8	134 ± 27.0	162.4 ± 32.7	21.2⇑
Yanci et al. [66]	New intervention	Plyometric training	Horizontal CMJ (cm)	8	2.0 ± 0.2	2.1 ± 0.2	5.0⇑	8	1.9 ± 0.1	2.0 ± 0.1	5.3⇑
Yanci et al. [66]	New intervention	Plyometric training	Horizontal CMJ with arms (cm)	8	2.4 ± 0.2	2.4 ± 0.3	0.0⇒	8	2.3 ± 0.1	2.3 ± 0.1	0.0⇒
*Vertical jumps*											
Chaabene et al. [71]	New intervention	Plyometric training	CMJ (cm)	13	23.66 ± 2.83	26.85 ± 2.78	13.5⇑	12	23.41 ± 5.55	26.47 ± 5.50	13.0⇑
Palma-Muñoz et al. [65]	New intervention	Plyometric training	CMJ (cm)	8	28.5 ± 10.4	31.4 ± 12.3	10.2⇑	7	28.4 ± 9.1	32.4 ± 7.2	14.1⇑
Palma-Muñoz et al. [65]	New intervention	Plyometric training	CMJ with arms (cm)	8	33.9 ± 11.1	35.2 ± 13.7	3.8⇑	7	34.0 ± 12.1	37.6 ± 8.0	10.6⇑
Ramírez-Campillo et al. [63]	New intervention	Plyometric training	CMJ (cm)	8	28.5 ± 6.9	31.5 ± 7.5	10.5⇑	8	27.4 ± 4.3	30.1 ± 4.7	9.9⇑
Ramírez-Campillo et al. [64]	New intervention	Plyometric training	CMJ with arms (cm)	8	28.5 ± 10.4	31.6 ± 11.5	10.9⇑	8	27.9 ± 8.7	32.5 ± 10.1	16.5⇑
Yanci et al. [66]	New intervention	Plyometric training	CMJ flight time (s)	8	0.55 ± 0.03	0.55 ± 0.05	0.0⇒	8	0.57 ± 0.04	0.54 ± 0.04	− 5.3⇓
Yanci et al. [66]	New intervention	Plyometric training	CMJ arm swing flight time (s)	8	0.55 ± 0.05	0.53 ± 0.05	− 3.6⇓	8	0.55 ± 0.03	0.51 ± 0.05	− 7.3⇓
Rebaï et al. [62]	Tapering after 4-week training¶	Resistance training program	CMJ (cm)	10	34.6 ± 5.3	36.8 ± 5.0	6.4⇑	10	35.4 ± 5.6	34.1 ± 5.5	− 3.7⇓
Hoffman et al. [59]	New intervention	Resistance training program	Vertical jump (cm)	15	65.9 ± 8.4	66.0 ± 8.8	0.2⇑	11	59.9 ± 6.7	62.5 ± 7.1	4.3⇑
Otero-Esquina et al. [70]	New intervention	Compound training, which encompasses strength training, plyometrics, and resisted sprinting	CMJ (cm)	12	34.48 ± 3.82	37.96 ± 2.74	10.1⇑	12	36.29 ± 4.21	39.32 ± 4.84	8.3⇑
Chaabene et al. [71]	New intervention	Plyometric training	SJ (cm)	13	21.29 ± 3.04	24.64 ± 2.84	15.7⇑	12	22.18 ± 5.00	25.64 ± 5.94	15.5⇑
Rebaï et al. [62]	Tapering after 4-week training¶	Resistance training program	SJ (cm)	10	32.2 ± 5.3	34.7 ± 5.1	7.8⇑	10	33.0 ± 5.5	32.6 ± 5.5	− 1.2⇓
Palma-Muñoz et al. [65]	New intervention	Plyometric training	20-cm drop jump (cm)	8	21.2 ± 6.9	28.2 ± 8.9	33.0⇑	7	22.5 ± 7.4	31.2 ± 8.9	38.7⇑
Ramírez-Campillo et al. [63]	New intervention	Plyometric training	20-cm drop jump (cm)	8	27.2 ± 5.9	30.9 ± 7.8	13.6⇑	8	27.7 ± 5.8	31.3 ± 6.6	13.1⇑
Ramírez-Campillo et al. [64]	New intervention	Plyometric training	20-cm drop jump RSI (mm/ms)	8	0.062 ± 0.022	0.0707 ± 0.0251	13.7⇑	8	0.072 ± 0.034	0.098 ± 0.046	36.1⇑
*Maximal strength*											
Hoffman et al. [59]	New intervention	Resistance training program	Squat (kg)	15	173.6 ± 36.2	186.3 ± 31.9	7.3⇑	11	191.6 ± 34.9	204.1 ± 39.5	6.5⇑
Naclerio et al. [68]	New intervention	Resistance training program (75%1RM)	Squat (kg)	6	103.0 ± 30.8	107.1 ± 30.6	4.0⇑	8	102.1 ± 26.7	119.8 ± 33.6	17.3⇑
Naclerio et al. [68]	New intervention	Resistance training program (75%1RM)	Bench press 1RM (kg)	6	49.3 ± 19.1	54.4 ± 22.1	10.3⇑	8	46.7 ± 19.6	54.5 ± 18.2	16.6⇑
Naclerio et al. [68]	New intervention	Resistance training program (75%1RM)	Upright row 1RM (kg)	6	40.8 ± 10.7	45.0 ± 13.8	10.3⇑	8	38.9 ± 10.7	45.7 ± 13.5	17.4⇑
Rebaï et al. [62]	Tapering after 4-week training	Resistance training program	Maximal voluntary contraction (N)	10	1071.8 ± 199.6	1168.1 ± 206.5	9.0⇑	10	1093.0 ± 199.5	1043.7 ± 173.7	− 4.5⇓
Severo-Silveira et al. [69]	New intervention	Eccentric training	Quadriceps concentric peak torque (N/m)	10	275.50 ± 27.17	276.60 ± 24.91	0.4⇑	11	278.05 ± 48.86	280.85 ± 53.95	1.0⇑
Severo-Silveira et al. [69]	New intervention	Eccentric training	Hamstrings concentric peak torque (N/m)	10	142.25 ± 19.66	144.74 ± 20.98	1.8⇑	11	146.64 ± 24.31	157.95 ± 30.48	7.7⇑
Severo-Silveira et al. [69]	New intervention	Eccentric training	Hamstrings eccentric peak torque (N/m)	10	204.58 ± 43.32	207.01 ± 41.67	1.2⇑	11	211.17 ± 31.81	225.64 ± 43.29	6.9⇑
Lacome et al. [67]#	New intervention	Eccentric training	Knee-flexor eccentric strength (N)	9	325 ± 26	362 ± 46	11.4⇑	10	326 ± 48	361.3	10.8⇑
*Sprint performance*											
Chaabene et al. [71]	New intervention	Plyometric training	Sprint 5 m (s)	13	1.19 ± 0.04	1.10 ± 0.06	− 7.6⇑	12	1.20 ± 0.10	1.16 ± 0.09	− 3.3⇑
Yanci et al. [66]	New intervention	Plyometric training	Sprint 5 m (s)	8	1.01 ± 0.05	1.02 ± 0.04	1.0⇓	8	1.01 ± 0.05	1.02 ± 0.06	1.0⇓
Bianchi et al. [5]	New intervention	Plyometric training	Sprint 10 m (s)	10	1.84 ± 0.08	1.79 ± 0.08	− 2.7⇑	11	1.85 ± 0.07	1.77 ± 0.08	− 4.3⇑
Chaabene et al. [71]	New intervention	Plyometric training	Sprint 10 m (s)	13	2.02 ± 0.05	1.94 ± 0.07	− 4.0⇑	12	2.10 ± 0.14	2.01 ± 0.13	− 4.3⇑
Palma-Muñoz et al. [65]	New intervention	Plyometric training	Sprint 10 m (s)	8	2.6 ± 0.3	2.6 ± 0.3	0.0⇒	7	2.7 ± 0.3	2.6 ± 0.2	− 3.7⇑
Ramírez-Campillo et al. [64]	New intervention	Plyometric training	Sprint 10 m (s)	8	2.64 ± 0.36	2.62 ± 0.36	− 0.8⇑	8	2.71 ± 0.29	2.67 ± 0.29	− 1.5⇑
Otero-Esquina et al. [70]	New intervention	Compound training, which encompasses strength training, plyometrics, and resisted sprinting	Sprint 10 m (s)	12	1.70 ± 0.06	1.70 ± 0.05	0.0⇒	12	1.71 ± 0.05	1.69 ± 0.05	− 1.2⇑
Ramírez-Campillo et al. [63]	New intervention	Plyometric training	Sprint 15 m (s)	8	3.28 ± 0.1	3.01 ± 0.1	− 8.2⇑	8	3.43 ± 0.1	3.10 ± 0.1	− 9.6⇑
Yanci et al. [66]	New intervention	Plyometric training	Sprint 15 m (s)	8	2.39 ± 0.10	2.39 ± 0.11	0.0⇒	8	2.40 ± 0.09	2.39 ± 0.07	− 0.4⇑
Otero-Esquina et al. [70]	New intervention	Compound training, which encompasses strength training, plyometrics, and resisted sprinting	Sprint 20 m (s)	12	2.99 ± 0.07	2.98 ± 0.08	− 0.3⇑	12	2.98 ± 0.09	2.93 ± 0.11	− 1.7⇑
Chaabene et al. [71]	New intervention	Plyometric training	Sprint 20 m (s)	13	3.50 ± 0.16	3.38 ± 0.13	− 3.4⇑	12	3.65 ± 0.24	3.54 ± 0.22	− 3.0⇑
Bianchi et al. [5]	New intervention	Plyometric training	Sprint 30 m (s)	10	4.25 ± 0.15	4.19 ± 0.15	− 1.4⇑	11	4.36 ± 0.16	4.26 ± 0.15	− 2.3⇑
Chaabene et al. [71]	New intervention	Plyometric training	Sprint 30 m (s)	13	4.98 ± 0.12	4.84 ± 0.17	− 2.8⇑	12	5.17 ± 0.34	5.03 ± 0.34	− 2.7⇑
Bianchi et al. [5]	New intervention	Plyometric training	Sprint 40 m (s)	10	5.48 ± 0.24	5.27 ± 0.27	− 3.8⇑	11	5.52 ± 0.18	5.46 ± 0.17	− 1.1⇑
Hoffman et al. [59]	New intervention	Resistance training program	40-yeard sprint (s)	15	5.01 ± 0.22	4.97 ± 0.18	− 0.8⇑	11	5.23 ± 0.20	5.18 ± 0.20	− 1.0⇑
*COD performance*											
Chaabene et al. [71]	New intervention	Plyometric training	T-test COD (s)	13	11.33 ± 0.34	10.54 ± 0.53	− 6.9⇑	12	11.58 ± 0.72	11.23 ± 0.77	− 3.0⇑
Palma-Muñoz et al. [65]	New intervention	Plyometric training	T-test COD (s)	8	13.0 ± 2.1	12.0 ± 1.9	− 7.7⇑	7	13.0 ± 1.5	11.8 ± 1.1	− 9.2⇑
Ramírez-Campillo et al. [64]	New intervention	Plyometric training	T-test COD (s)	8	13.0 ± 2.1	12.0 ± 1.9	− 7.7⇑	8	13.1 ± 1.5	11.9 ± 1.4	− 9.2⇑
Bianchi et al. [5]	New intervention	Plyometric training	5–0-5 COD test (s)	10	4.78 ± 0.12	4.69 ± 0.17	− 1.9⇑	11	4.83 ± 0.16	4.73 ± 0.16	− 2.1⇑
Ramírez-Campillo et al. [63]	New intervention	Plyometric training	Meylan COD (s)	8	4.94 ± 0.2	4.57 ± 0.2	− 7.5⇑	8	5.12 ± 0.3	4.74 ± 0.3	− 7.4⇑
Yanci et al. [66]	New intervention	Plyometric training	Modified agility test COD (s)	8	4.92 ± 0.22	4.86 ± 0.25	− 1.2⇑	8	4.87 ± 0.25	4.87 ± 0.20	0.0⇒
Otero-Esquina et al. [70]	New intervention	Compound training, which encompasses strength training, plyometrics, and resisted sprinting	V-cut COD test (s)	12	6.66 ± 0.20	6.51 ± 0.18	− 2.3⇑	12	6.80 ± 0.42	6.46 ± 0.25	− 5.0⇑
*Aerobic performance*											
Ramírez-Campillo et al. [63]	New intervention	Plyometric training	YYIRT level 1(m)	8	573 ± 237	628 ± 244	9.6⇑	8	630 ± 192	690 ± 203	9.5⇑
Ramírez-Campillo et al. [64]	New intervention	Plyometric training	YYIRT level 1 (m)	8	990 ± 440	1105 ± 491	11.6⇑	8	993 ± 457	1145 ± 527	15.3⇑
Hoffman et al. [59]	New intervention	Resistance training program	2-mile run (s)	15	945.0 ± 61.3	830.7 ± 55.5	− 12.0⇑	11	982.2 ± 65.0	879.8 ± 68.7	− 10.4⇑

⇑: improvement from post–pre (within-group difference); ⇓decline from post–pre (within-group difference); ⇒: maintenance from post–pre (within-group difference); CMJ: countermovement jump; SJ: squat jump; #: collected from pre-to-mid-test, since after was crossover; RM: repetition maximum; COD: change-of-direction; YYIRT: Yo-Yo Intermittent recovery test; RSI: reactive strength index; ¶ with both groups exposed to Ramadan fasting; LV: lower-volume training; HV: higher-volume training

However, in terms of strength-related outcomes, contradictory findings have emerged. Some studies, such as those by Hoffman et al. [59] and Lacome et al. [67], reported similar results for lower-volume and higher-volume training, with improvements of 7.3% and 11.4% for lower-volume training and 6.5% and 10.8% for higher-volume training, respectively. In contrast, Naclerio et al. [68] and Severo-Silveira et al. [69] observed adaptations of 4.0% and 1.8% for lower-volume training and 17.3% and 7.7% for higher-volume training.

While improvements in change of direction (COD) were similar between lower-volume training (ranging from 1.9% [5] and 7.7% [65]) and higher-volume training (ranging from 2.1% [5] and 9.2% [65]) across the studies reviewed, the evidence regarding 10-m linear sprint times was contradictory. Both Bianchi et al. [5] and Chaabene et al. [71] reported comparable percentages of improvement, whereas Palma-Muñoz et al. [65] found a significant advantage for higher-volume training.

Regarding aerobic performance, Ramírez-Campillo et al. [63] and [64] found similar improvements in both lower- and higher-volume plyometric training groups. Similarly, Hoffman et al. [59] reported comparable improvements following resistance training.

Table 8 summarizes individual studies on running-based training. Shi et al. [18] reported that the lower-volume training group improved by 3.8%, 3.5%, and 18.2% in maximal oxygen uptake, maximal sprint velocity, and Yo-Yo intermittent recovery test level 1, respectively, while the higher-volume training group showed improvements of 1.3%, 0.4%, and 21.7%, respectively. In the context of tapering strategies, Krespi et al. [58] found that lower-volume training outperformed higher-volume training, yielding improvements of 4.9% versus 0.5% in the 10-m sprint, 1.9% versus 0.5% in the countermovement jump, and 1.6% versus 0.8% in maximal oxygen uptake.

**Table 8:** Results of individual studies—running-based training

Study	Context	Training type	Variable	LV (n)	LV pre	LV post	% (post–pre)	HV (n)	HV pre	HV post	% (post–pre)
Krespi et al. [58]	Tapering after 4-week training	Running exercises (90–95%HRmax)	Sprint 5 m (s)	79	1.13 ± 0.15	1.02 ± 0.17	− 9.7⇑	79	1.15 ± 0.17	1.06 ± 0.17	− 7.8⇑
Krespi et al. [58]	Tapering after 4-week training	Running exercises (90–95%HRmax)	Sprint 10 m (s)	79	2.04 ± 0.13	1.94 ± 0.16	− 4.9⇑	79	1.98 ± 0.17	1.97 ± 0.18	− 0.5⇑
Krespi et al. [58]	Tapering after 4-week training	Running exercises (90–95%HRmax)	Sprint 30 m (s)	79	4.34 ± 0.22	4.25 ± 0.24	− 2.1⇑	79	4.25 ± 0.20	4.22 ± 0.18	− 0.7⇑
Krespi et al. [58]	Tapering after 4-week training	Running exercises (90–95%HRmax)	COD test with 180º (s)	79	7.46 ± 0.36	7.41 ± 0.29	− 0.7⇑	79	7.48 ± 0.44	7.41 ± 0.40	− 0.9⇑
Krespi et al. [58]	Tapering after 4-week training	Running exercises (90–95%HRmax)	RSA (s)	79	6.90 ± 0.38	6.74 ± 0.37	− 2.3⇑	79	6.88 ± 0.44	6.73 ± 0.45	− 2.2⇑
Krespi et al. [58]	Tapering after 4-week training	Running exercises (90–95%HRmax)	SJ (cm)	79	42.06 ± 4.41	42.77 ± 4.65	1.7⇑	79	41.68 ± 4.24	42.07 ± 4.45	0.9⇑
Krespi et al. [58]	Tapering after 4-week training	Running exercises (90–95%HRmax)	CMJ (cm)	79	53.69 ± 5.36	54.73 ± 5.09	1.9⇑	79	51.77 ± 5.12	52.05 ± 5.19	0.5⇑
Krespi et al. [58]	Tapering after 4-week training	Running exercises (90–95%HRmax)	VO2max (ml/kg/min)	79	56.98 ± 3.89	57.88 ± 4.10	1.6⇑	79	56.03 ± 5.56	56.48 ± 5.66	0.8⇑
Shi et al. [18]	New intervention	Repeated sprint training in hypoxia	VO2max (ml/kg/min)	10	58.4 ± 2.9	60.6 ± 3.6	3.8⇑	10	62.8 ± 3.9	63.6 ± 3.5	1.3⇑
Shi et al. [18]	New intervention	Repeated sprint training in hypoxia	Maximal sprint velocity (m/s)	10	5.10 ± 0.24	5.28 ± 0.22	3.5⇑	10	5.08 ± 0.27	5.10 ± 0.16	0.4⇑
Shi et al. [18]	New intervention	Repeated sprint training in hypoxia	YYIRT level 1 (m)	10	1340 ± 172	1584 ± 287	18.2⇑	10	1404 ± 322	1708 ± 362	21.7⇑

⇑, improvement from post–pre (within-group difference); COD, change-of-direction; RSA, repeated sprint ability; CMJ, countermovement jump; SJ, squat jump; VO2max, maximal oxygen uptake; LV, lower-volume training; HV, higher-volume training

Table 9 summarizes studies on mixed training (e.g., in-field training combined with strength training). Beltran-Valls et al. [60] found that lower-volume training improved vertical countermovement jump, 10-m sprint, and Illinois change-of-direction time by 5.3%, 2.9%, and 1.3%, respectively, while higher-volume training showed only 0.5%, 1.2%, and 0.4% improvements. Additionally, another study [61] revealed that lower-volume training led to a 5.2% increase in maximal oxygen uptake and an 18.9% improvement in the Yo-Yo intermittent recovery test level 1 after 8 weeks. Coutts et al. [57] reported that the lower-volume group improved maximal oxygen uptake by 0.8% and vertical jump by 0.3%, while peak cycling power decreased by 3.0%. In contrast, the higher-volume group experienced a 7.7% decline in maximal oxygen uptake, a 4.1% decline in countermovement jump, and a 3.2% decline in peak cycling power.

**Table 9:** Results of individual studies—mixed training

Study	Context	Training type	Variable	LV (n)	LV pre	LV post	% (post–pre)	HV (n)	HV pre	HV post	% (post–pre)
Beltran-Valls et al. [60]	Tapering after 6-week training	Plyometrics, COD and SSGs	CMJ (W/kg)	11	1029.71 ± 108.51	1084.21 ± 110.87	5.3⇑	11	945.31 ± 98.72	949.99 ± 113.32	0.5⇑
Beltran-Valls et al. [60]	Tapering after 6-week training	Plyometrics, COD and SSGs	Sprint 10 m (s)	11	1.72 ± 0.09	1.67 ± 0.07	− 2.9⇑	11	1.72 ± 0.06	1.70 ± 0.06	− 1.2⇑
Beltran-Valls et al. [60]	Tapering after 6-week training	Plyometrics, COD and SSGs	Illinois COD (s)	11	15.39 ± 0.35	15.19 ± 0.16	− 1.3⇑	11	15.78 ± 0.37	15.72 ± 0.41	− 0.4⇑
Coutts et al. [57]	New intervention	Field sessions and resistance training	VO2max (ml/kg/min)	9	51.2 ± 4.8	51.6 ± 3.5	0.8⇑	9	51.9 ± 2.5	47.9 ± 3.7	− 7.7⇓
Coutts et al. [57]	New intervention	Field sessions and resistance training	Vertical jump (cm)	9	60.2 ± 4.5	60.4 ± 8.0	0.3⇑	9	63.7 ± 11.3	61.1 ± 11.3	− 4.1⇓
Coutts et al. [57]	New intervention	Field sessions and resistance training	Peak cycling power (W)	9	1027 ± 80	996 ± 104	− 3.0⇓	9	1032 ± 52	999 ± 84	− 3.2⇓
Fortes et al. [61]	Tapering after 8-week training	Physical, technical and tactical training	VO2max (ml/kg/min)	47	50.28 ± 2.68	52.90 ± 2.26	5.2⇑	47	50.11 ± 2.40	50.39 ± 2.49	0.6⇑
Fortes et al. [61]	Tapering after 8-week training	Physical, technical and tactical training	YYIRT level 1 (m)	47	1652.38 ± 96.29	1964.28 ± 114.38	18.9⇑	47	1632.14 ± 95.04	1665.47 ± 100.82	2.0⇑

⇑, improvement from post–pre (within-group difference); ⇓, decline from post–pre (within-group difference); COD, change-of-direction; LV, lower-volume training; HV, higher-volume training; VO2max, maximal oxygen uptake; YYIRT, Yo-Yo Intermittent recovery test

### Meta-analysis

**Fig. 2:**
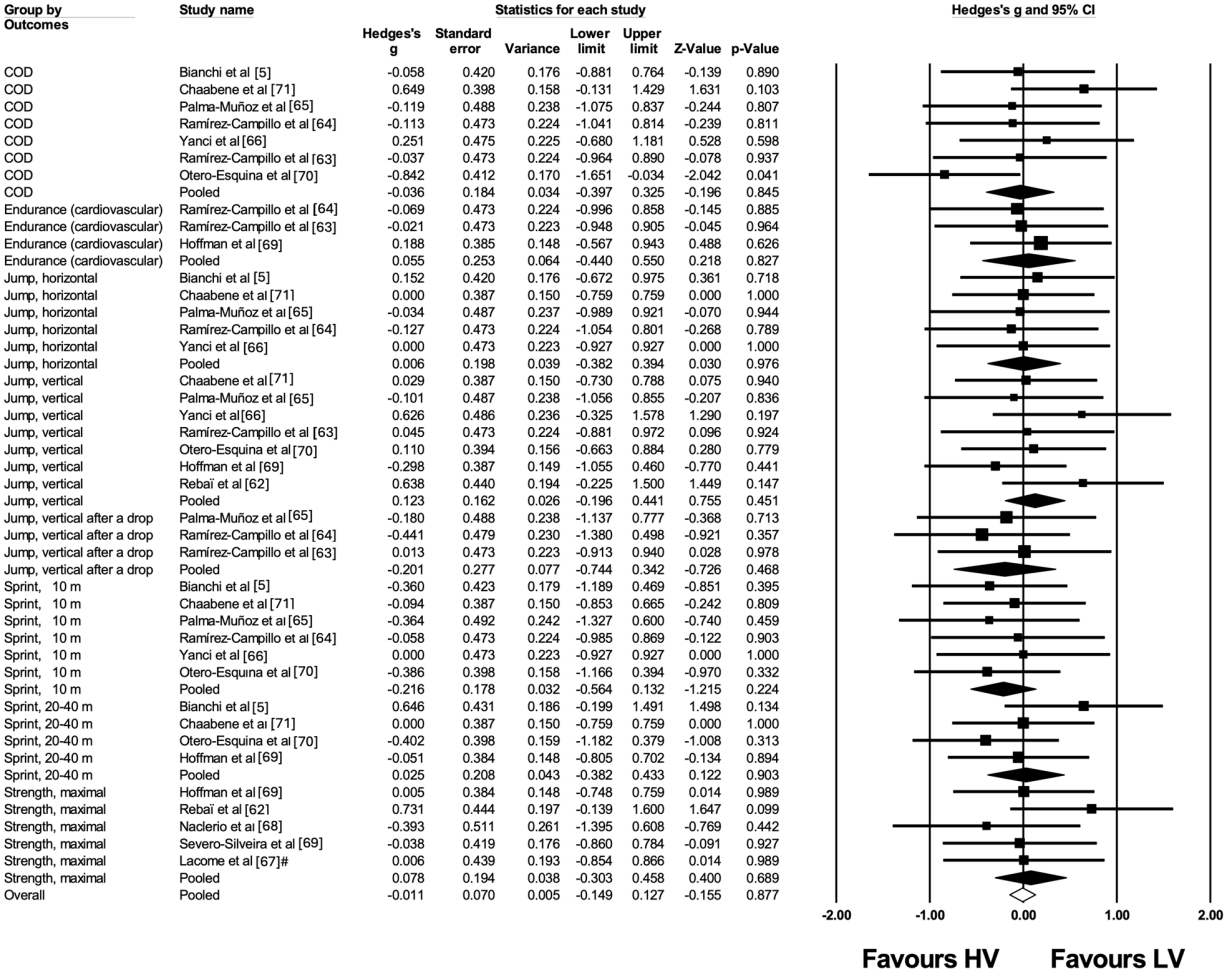
Forest plot illustrating changes in physical fitness outcomes after higher-volume in comparison to lower-volume resistance training interventions. Forest plot values are shown as effect sizes (ES [Hedges’ g]) with 95% confidence intervals (CI). Black squares: individual studies. White rhomboid: overall summary value. Black rhomboid: summary value for each physical fitness outcome. *, &, $, and %: denotes that repeated studies, but with different symbols, provided ≥ 1 outcome to the analyses; #: collected from pre-to-mid-test, since after was crossover; LV: lower-volume training; HV: higher-volume training; COD: change-of-direction

The results (Fig. 2) showed a non-significant difference between the higher-volume compared to the lower-volume training groups in the overall ES (all physical fitness outcomes) in resistance-based training = − 0.05, 95% CI − 0.19 to 0.09, *p* = 0.506, *I*^2^ = 0.0%, total participants *n* = 213, Egger's test two-tailed = 0.819. To avoid bias in the overall ES due to the inclusion of two or more outcomes from a single study, outcome-specific analyses were considered. Specifically, the results (Fig. 2) showed non-significant (all physical fitness outcomes *p* > 0.05) differences between the higher-volume compared to the lower-volume training groups in resistance training for COD speed (ES = − 0.04, 95% CI = − 0.40 to 0.33, *p* = 0.845, and *I*^2^ = 16.7%), cardiorespiratory endurance (ES = 0.06, 95% CI = − 0.44 to 0.55, *p* = 0.827, and *I*^2^ = 0.0%), horizontal jump distance (ES = 0.01, 95% CI = − 0.38 to 0.39, *p* = 0.976, and *I*^2^ = 0.0%), vertical jump height (ES = 0.04, 95% CI = − 0.30 to 0.38, *p* = 0.813, and *I*^2^ = 0.0%), reactive strength (ES = − 0.20, 95% CI = − 0.74 to 0.34, *p* = 0.468, and *I*^2^ = 0.0%), maximal strength (ES = − 0.08, 95% CI = − 0.50 to 0.35, *p* = 0.721, and *I*^2^ = 0.0%), 10-m sprinting performance (ES = − 0.22, 95% CI = − 0.56 to 0.13, *p* = 0.224, and *I*^2^ = 0.0%), or 20- 40-m sprinting performance (ES = 0.03, 95% CI = − 0.38 to 0.43, *p* = 0.903, and *I*^2^ = 8.0%).

### Certainty of Evidence

Table 10 illustrates the certainty assessment conducted through GRADE analysis. It is crucial to emphasize that the certainty of evidence pertaining to physical performance outcomes was ascertained to be very low. This was predominantly attributed to the substantial risk of bias identified across most of the encompassed studies. Additionally, the imprecision in the reported effects on physical performance, arising from the limited number of participants, further diminished the certainty of evidence. The diminished sample sizes, coupled with the absence of a clear direction of effects in the comparative analyses between lower-volume and higher-volume training groups, collectively contributed to the very low level of certainty of the evidence.

**Table 10:** GRADE analysis

Outcomes (LV vs HV)	Studies and PSS	Risk of bias in studies	Risk of publication bias	Inconsistency	Imprecision	Certainty of evidence
COD	7, n = 133	Downgrade by two levels (high-risk of bias)	Not applicable	No downgrading (*I*^2^ = 16.7%)	Downgrade by two levels: (i) < 800 participants; (ii) no clear direction of effect	⊕, Very low
Endurance (cardiovascular)	3, n = 58	Downgrade by two levels (high-risk of bias)	Not applicable	No downgrading (*I*^2^ < 0.01%)	Downgrade by two level: (i) < 800 participants; (ii) no clear direction of effect	⊕, Very low
Horizontal jump	4, n = 72	Downgrade by two levels (high-risk of bias)	Not applicable	No downgrading (*I*^2^ < 0.01%)	Downgrade by two levels: (i) < 800 participants; (ii) no clear direction of effect	⊕, Very low
Vertical jump	7, n = 142	Downgrade by two levels (high-risk of bias)	Not applicable	No downgrading (*I*^2^ < 0.01%)	Downgrade by two level: (i) < 800 participants; (ii) no clear direction of effect	⊕, Very low
Drop jump	3, n = 47	Downgrade by two levels (high-risk of bias)	Not applicable	No downgrading (*I*^2^ < 0.01%)	Downgrade by two levels: (i) < 800 participants; (ii) no clear direction of effect	⊕, Very low
Sprint 10-m	6, n = 117	Downgrade by two levels (high-risk of bias)	Not applicable	No downgrading (*I*^2^ < 0.01%)	Downgrade by two level: (i) < 800 participants; (ii) no clear direction of effect	⊕, Very low
Sprint 20–40 m	4, n = 96	Downgrade by two levels (high-risk of bias)	Not applicable	No downgrading (*I*^2^ = 8.04%)	Downgrade by two levels: (i) < 800 participants; (ii) no clear direction of effect	⊕, Very low
Maximal strength	5, n = 100	Downgrade by two levels (high-risk of bias)	Not applicable	No downgrading (*I*^2^ < 0.01%)	Downgrade by two level: (i) < 800 participants; (ii) no clear direction of effect	⊕, Very low

(i) *Risk of bias in studies*: downgraded by one level if some concerns and two levels if high-risk of bias; (ii) *Indirectness*: considered low due to eligibility criteria; (iii) *Risk of publication bias*: not assessed, as all comparison had < 10 studies available; downgrade one level if Egger’s test < 0.05; (iv) *Inconsistency*: downgraded by one level when the impact of statistical heterogeneity (*I*^2^) was moderate (> 25%) and by two levels when high (> 75%); (v) *Imprecision*: downgraded by one level when < 800 participants were available for a comparison or if there was no clear direction of the effects [89]; accumulation of both resulted in downgrading by two levels

GRADE, Grading of Recommendations Assessment, Development and Evaluation; LV, lower-volume training; HV, higher-volume training; PSS, pooled sample size

## Discussion

When facing schedule congestion and the challenges associated with implementing effective training, coaches in team sports are required to adopt a nuanced and intricate approach to designing sessions. Such an approach should prioritize the provision of the necessary stimuli for improvement while minimizing the impact on players’ physical readiness. With this concept in mind, this systematic review and meta-analysis explored experimental studies comparing training approaches, specifically examining the differences between lower-volume and higher-volume training in team sports players.

Our analysis revealed a greater focus on resistance-based training compared to aerobic-based training, with plyometric training being the most commonly used approach, followed by traditional resistance training and eccentric-based training. Regarding resistance-based training, our meta-analysis focused on the physical performance adaptations conferred by that specific modality. Interestingly, the results revealed that lower-volume training yielded comparable results to higher-volume training, suggesting that both approaches have similar effects on key physical performance variables such as vertical jump, horizontal jump, change-of-direction ability, linear speed over 10 m and between 20 and 40 m, maximal strength, and aerobic performance.

Conversely, interventions focused on aerobic-based training (e.g., running) or studies that considered in-field training volume as a factor, or that combined resistance training with aerobic training, were primarily analyzed in the context of tapering strategies (i.e., comparing higher versus lower reductions in training volume). This contrasts with most resistance-based training studies in our review, which centered on newly introduced training interventions. It is also important to emphasize that the current review aimed to understand the impact of lower versus higher training volumes. This included studies that introduced new interventions to maximize adaptations, as well as other studies focused on tapering strategies, which aim to decrease training volume to promote supercompensation. Although the strategies and goals are considerably different, both approaches utilize training volume as part of their methodological context to achieve positive adaptations.

While our analysis of lower versus higher training volumes in resistance training indicated that newly introduced training interventions tend to yield similar adaptations, with no significant differences in physical fitness outcomes, the context of tapering revealed different tendencies. Although we were unable to conduct a meta-analysis due to insufficient data, the included studies suggested that a lower volume (i.e., a higher taper) may lead to a more favorable tendency for enhancing physical fitness adaptations through supercompensation. However, the limited data prevented us from conclusively confirming this trend.

### The Effects of Lower-Volume Versus Higher-Volume Resistance-Based Training on Physical Performance of Team Sports Players

Resistance-based training, as it applies to team sports, is commonly regarded as a supplementary strategy aimed at elevating athletes’ physical and sports performance [72] while mitigating the risk of injuries [73]. Among the available options, plyometrics stands out as one of the most widely adopted [74] owing to its proven efficacy and straightforward application. This prevalence is evident in the resistance-based training studies encompassed in our systematic review, in which plyometrics emerged as the predominant training method as in the studies of Bianchi et al. [5], Chaabene et al. [71], Palma-Muñoz et al. [65], Ramírez-Campillo et al. [64], and Yanci et al. [66]. Furthermore, the specific focus on eccentric training emerged as another noteworthy aspect of interest within the studies included in this systematic review, as in the cases of Severo-Silveira et al. [69] and Lacome et al. [67].

Depending on the adopted method, resistance-based training can enhance the neuromuscular readiness of players [75], which can influence their capacity to demonstrate high performance during in-field tactical and technical training. Accordingly, strength and conditioning coaches must achieve a fine-tuned balance of training activities, which involves the strategic minimization of training volume while striving to maximize training effectiveness [76]. Traditionally, these goals may have been considered to conflict with each other, but our results suggest that this might not be the case in practice.

An examination of the included studies revealed that newly-introduced resistance-based training programs that exposed athletes to novel training stimuli (either as a substitute for or an addition to the regular training regimen) and employing either lower- or higher-volume training (ranging from + 1.3 times more than the lower-volume, as seen in the case of Hoffman et al. [59], up to + 4 times more, as seen in the case of Lacome et al. [67]) generally has similar effects on physical performance. While we acknowledge the heterogeneity in overall training volume and the differences between lower and higher training volumes—factors that complicate the classification of training doses and prevent us from making definitive statements regarding optimal dosages—we can observe a tendency in the results indicating that adaptations can be similar at volumes ranging from more than 1 to up to 4 times greater. This similarity may be attributed to regular in-field training sessions to which players are also exposed, as well as the overall context of each study. For example, in the study by Lacome et al. [67], the lower volume consisted of 2 sets of eccentric training in a single weekly session, compared to 8 sets in the same condition. On the other hand, Hoffman et al. [59] reported a lower training volume of 3 weekly sessions versus a higher volume of 6 weekly sessions. In this regard, our review does not aim to identify a minimal effective dose, as such determinations are closely associated with specific sports contexts. For instance, in soccer, introducing just 1 session with 2 sets was sufficient to ensure adaptations, whereas 8 sets represented a higher volume. Conversely, in American Football, both approaches introduced in soccer might be considered very small doses given their cultural emphasis on strength training. Thus, rather than establishing a minimal or ideal low dose—which is not feasible due to the scarcity of evidence across various sports and the heterogeneity of populations—our results aim to highlight patterns that hold across diverse scenarios comparing lower and higher training volumes.

For instance, Bianchi et al. [5] and Chaabene et al. [71] both incorporated plyometric training in young soccer players and demonstrated a roughly two-fold difference between higher-volume and lower-volume training. Notably, both studies reported noteworthy within-group enhancements of vertical and horizontal jumping performance, as well as linear sprint speed. Remarkably, no significant differences in performance were observed between the groups who undertook programs of different training volumes.

Similar to the above, Ramírez-Campillo et al. [64] identified analogous trends when applying plyometric training methods to young soccer players. The researchers reported improvements in jumping performance, change-of-direction ability, and sprinting for both lower- and higher-volume training protocols. Furthermore, significant enhancements in cardiorespiratory performance were found with both training volumes, with no significant differences noted between groups. The findings suggest that the specific physical demands of team sports can be effectively addressed with lower training frequencies, specifically one to two training sessions per week. This approach demonstrates efficacy in yielding significant improvements in lower-limb power, speed, and endurance. The intensity of the stimulus that underscores neural drive, changes in muscle activation, stretch–shortening cycle activity, and stiffness in the lower limbs may provide a rationale for the observed effectiveness [77]. However, Ramírez-Campillo et al. [64] made a noteworthy finding that higher-volume training appeared to be significantly more effective than lower-volume training in enhancing maximal kicking velocity, a critical soccer-specific performance metric. This finding warrants further investigation, particularly regarding the integration with technical skill training to optimize improvements.

However, the specific reasons for the lack of differentiation between lower-volume and higher-volume training remain unknown. Emerging evidence suggests that the anabolic signaling associated with the mechanical tension placed on the involved musculature is responsible [78]. Perhaps the first few repetitions of a set can provide a greater benefit than the last repetitions, indicating why higher volumes may be less effective than commonly perceived—later repetitions are less impactful, resulting in diminishing returns per repetition. However, further research is necessary to confirm this, and additional studies elucidating causality are still required. [79].

Eccentric training constitutes another major focal point within the included studies, with Severo-Silveira et al. [69] and Lacome et al. [67] revealing variations in training volume differences. Severo-Silveira et al. [69] emphasized that a progressive training periodization, characterized by higher-volume training, demonstrated a potential advantage in improving both concentric and eccentric strength in the hamstrings. This effect suggests a potential for increased adaptability to stimuli, particularly from the heightened exposure to eccentric forces. Moreover, higher volume was more likely to target the long head fascicle of biceps femoris compared to the constant training group, which also utilized lower-volume training.

Conversely, Lacome et al. [67] implemented a crossover design wherein both groups were introduced to eccentric training for the first six weeks of the study. Following a one-week washout period, they switched to the opposite groups for the next six weeks. The results demonstrated that lower-volume training was equally effective as higher-volume training in improving knee-flexor strength and fascicle length.

While research on underlying mechanisms remains limited, it appears that the repetitions within a session may not particularly significantly improve physical performance and morphological changes. For novices to eccentric training, even small doses of this activity can elicit pronounced improvements in muscle function and performance, as the novel stimulus challenges the neuromuscular system and promotes rapid adaptations as greater mechanical tension and muscle damage are induced compared to concentric contractions [80, 81].

Interestingly, the specific design outlined by Lacome et al. [67] revealed a plateau in fascicle lengthening after six weeks of training. This observation aligns with previous findings, such as those showing a reduction of additional lengthening in vastus lateralis after five weeks of isokinetic eccentric training [82]. This implies a potential ceiling effect in fascicle lengthening induced by eccentric training, underscoring the need for further analysis on manipulating the variation of load (progressivity and undulation periodization) and intensity (e.g., load, tempo, range of motion) in experimental studies of a longer duration.

The timing of training within the sport season appears to be another crucial aspect that is sensitive to load accommodation and adaptation. For instance, Naclerio et al. [68] demonstrated that a higher-volume protocol was more effective than a lower-volume approach for enhancing maximum strength. In contrast, lower-volume protocols emerged as preferable strategies for improving lower-body or upper-body average power performance, respectively, in collegiate team sport athletes with no prior resistance training experience. The authors recommended incorporating higher-volume resistance training protocols during the early phase of training to facilitate team sport athletes, particularly those with no prior resistance training experience, to increase strength performance in a relatively short period. Subsequently, they suggested transitioning to lower-volume protocols to help maintain the strength gained throughout the season.

Taken together, the findings regarding resistance-based training in team sports suggest that, when contextualized to specific teams and populations, lower training volumes can be as effective as higher volumes in achieving key physical fitness outcomes. This may be attributed to the significant contribution of in-field training sessions to athletes' overall training regimens. As a result, athletes can experience positive adaptations with fewer repetitions or weekly sessions in the context of resistance training. However, it is crucial to consider the cultural approach to strength training specific to each sport, as well as the scheduling constraints and athlete types. Therefore, definitive conclusions regarding the ideal low or minimal effective dose remain elusive and warrant further research in team sports athletes.

### The Effects of Lower-Volume Versus Higher-Volume Training Under Specific Periodization

Among the studies with an aim other than strength training, there was a smaller number of research articles, and those that did exist were much more heterogeneous. This heterogeneity was the reason for not conducting a meta-analysis. Specifically, it was noted that the studies integrated were not only those comparing lower- versus higher-volume training but also included specific types of periodization, such as tapering, with different training volumes in running-based and mixed-based training interventions.

Shi et al. [18] conducted an experimental study involving university athletes participating in football and handball. The study implemented a two-week repeated sprint training regimen under hypoxia conditions versus a group undergoing a five-week repeated sprint training program in hypoxia. In this case, the difference in training volume was attributed to the varying number of training sessions; thus, a higher number of sessions resulted in a greater overall training volume. Those in the hypoxic group showed significant enhancements within the first 2 weeks. For participants in the 5-week hypoxic training program, the immediate improvement in repeated sprint ability after training was comparable to that in the 2-week program. Notably, the positive effects of the 5-week hypoxic training were well-sustained four weeks after the program was completed, indicating enduring benefits in repeated sprint ability.

One limitation we identified pertains to the heterogeneity among studies on running-based and mixed-based programs. Specifically, we encountered a distinctive type of comparison that focused on varying volumes during the tapering phases. Despite being influenced by the preceding volume, we incorporated studies with similar volumes before tapering, wherein divergent volumes were exclusively compared within the tapering phase. Coutts et al. [57] examined overreaching in rugby players subjected to intense training. One group underwent 6 weeks of regular training, while the other intentionally experienced overreaching with intensified training (1.2 times the normal load) [57]. The findings indicated that after 6 weeks of intensity training, aerobic performance and maximal oxygen uptake decreased significantly more in the higher-volume training group compared to the lower-volume control group [57]. Intriguingly, a brief taper led to supercompensation in aerobic performance, increased vertical jump height, maximal oxygen uptake, reduced muscle damage, and a return to a more anabolic hormonal environment in the higher-volume training group [57].

Tapering involves a purposeful reduction in training duration and frequency as athletes approach a competition or a designated peak performance phase [83]. The primary goals of tapering are to facilitate recovery from the accumulated fatigue resulting from intense training, optimize both physiological and psychological readiness, and ultimately enhance performance during the competition [84]. There are various approaches to tapering, with discussions centered around determining the optimal magnitude of decreases in load to effectively leverage the supercompensation curve [83, 85].

From the included studies, we identified tapering strategies that involved a comparison between volume training during specific periods. Such strategies were observed in mixed-based training studies, as exemplified by Beltran-Valls et al. [60] and Fortes et al. [61]. Additionally, tapering strategies were observed in running-based training, as seen in the study conducted by Krespi et al. [58]. Furthermore, Rebaï et al. [62] explored tapering approaches in resistance-based training.

Regarding mixed training, Beltran-Valls et al. [60] compared tapering (with a reduction of 2.1 times in training load while maintaining the same intensity) against continuing with a regular training load in soccer players over 2 weeks. The outcomes of this study [60] indicated that tapering improved lower-limb muscle power and acceleration capacities, accompanied by a reduction in the stress state when compared to the control group.

Similarly, following a comparable design approach, Fortes et al. [61] conducted a study comparing a three-week tapering strategy (involving a reduction of 20–60% in training volume) against maintaining the regular training load in soccer players. The findings demonstrated a significant enhancement in maximal oxygen uptake with the tapering approach compared to the control group. The control group, in this instance, adhered to a higher-volume training regimen and exhibited no significant change in maximal oxygen uptake.

Indeed, the duration of tapers, ranging from eight to 14 days, appears to be a critical threshold according to which the favorable effects of tapering can potentially transition into detrimental effects associated with detraining [83]. Tapers can vary in length from one to three weeks. The sensitivity of these effects is contingent upon individual athlete factors and the tapering strategy employed, particularly the magnitude of load reduction and its progression.

For instance, Krespi et al. [58] using running-based training tested two tapering approaches in soccer players. One group experienced a linear reduction in load (4 × 4 min in week one, 3 × 4 min in week two, 3 × 4 min in week three, and finally 1 × 4 min in week four), while the other group underwent an exponential reduction (4 × 4, 2 × 4, 1 × 4, and 1 × 4 min, respectively). The results demonstrated that exposure to exponential tapering had significantly better effects on speed (linear sprint), countermovement jump, and maximal oxygen uptake. Tapering strategies have favorable effects on blood markers, such as erythrocyte, hemoglobin, and hematocrit volume, as well as testosterone. They also improve muscle glycogen content [86], which can justify multiple improvements from endurance to neuromuscular outcomes in parallel with and optimized oxygen extraction [86] and myosin-heavy chain IIA isoforms by increasing fiber cross-sectional area, peak force, and power output [86–88].

Furthermore, tapering strategies have been associated with favorable effects on blood markers such as erythrocyte count, hemoglobin levels, hematocrit volume, and testosterone. Additionally, these strategies have shown improvements in muscle glycogen content, coinciding with optimized oxygen extraction, a greater proportion of fast myosin-heavy chains, and a shift toward type IIa fibers. This physiological shift can justify multiple improvements, ranging from endurance to neuromuscular outcomes.

### Research Limitations and Future Research

The present systematic review is not without limitations. Most studies on this topic rely on small sample sizes and often lack a priori sample size estimations. This limitation undermines the generalizability of individual studies and can also lead to insufficient statistical power in our systematic review, even when the data are pooled. Additionally, we observed a sex-related publication bias, with a noted predominance of studies conducted in men. This creates a gap in the current understanding of the potential impacts and responses in women. Another important limitation arises from the variability in training load reductions and volumes among the studies included in the meta-analysis, which introduces methodological differences. Furthermore, the data collected for modalities other than resistance-based training were relatively sparse, potentially leading to an imbalanced interpretation of the results.

The diversity in population age, types of sports, and training status can lead to heterogeneity in the interpretation of results. Additionally, since these studies focus on team sports that already incorporate in-field training sessions, it becomes practically challenging to isolate the effects of newly introduced training programs from those of existing training sessions, particularly in-field activities. Often, these new programs contribute only one element among many, making it challenging to discern the specific effects. The inability to isolate mechanisms, sensitivity, and responsiveness to a given dose makes it difficult to establish causal effects. Consequently, the recommended or effective dose remains indefinable for a specific training program, ensuring minimal effective adaptation and identifying the optimal point at which individuals can derive maximum benefits from volume increases.

Another limitation of this review is the practical challenge posed by the comparisons between lower and higher volume training across the individual studies. These comparisons are fundamentally different due to the heterogeneity in overall training volume, intensity, and session frequency. As a result, the lower volume in one study may represent a higher volume in another. This can be seen as a barrier to the effective interpretation of our findings. This variability makes it impractical to establish a solid identification of an “ideal” dose for players. However, acknowledging this limitation is crucial for addressing the a priori research question: “Are there differences in the magnitude of adaptations in physical fitness between athletes exposed to lower versus higher training volumes?” This indicates that our focus is on identifying the impact of varying training volumes rather than pinpointing a specific minimal or ideal dose. Thus, comparing lower versus higher volumes becomes a somewhat “reductionist” approach, as the precise load is contingent on partial increases or decreases tailored to an individual’s adaptation threshold—an aspect that has not been thoroughly analyzed. Future research should delve into individual adaptation thresholds by applying a combination of assessments and control measures for factors related to load, recovery, and individual trainability. A nuanced understanding of these factors is essential for determining the optimal minimally effective dose that remains as potent as other potentially adjustable training regimens tailored to specific players.

### Practical Applications/Implications

While the optimal or minimal effective dose for sports athletes remains unclear, largely due to individual variability and responsiveness to training stimuli, which are significantly influenced by factors such as trainability, genetics, season phase, recovery strategies, and more, our research suggests that although no ideal training volume can be definitively prescribed, some conclusions can be drawn. Specifically, in resistance training for athletes, one weekly session can yield similar adaptations in jumping performance, change of direction, and cardiorespiratory endurance when compared to two weekly sessions. Similarly, when comparing groups training twice a week, similar adaptations can be observed in those performing half the overall training volume per session.

It remains challenging to recommend an exact training dose due to the heterogeneity of study designs and comparisons, making it difficult to offer a universal prescription. However, it is important to acknowledge that, in team sports, additional interventions—whether in resistance or aerobic-based training—can produce similar results with lower volumes when comparing to higher. It is up to coaches to determine whether increasing the training load of an athlete’s strength and conditioning program is necessary, especially when field training already provides a substantial and multifactorial stimulus.

Additionally, our study examined different training volumes in two contexts: when a new training intervention was introduced and during tapering (i.e., reducing training volume after previous weeks of training). The results suggest that tapering with lower volumes can be particularly beneficial, supporting the concept of supercompensation.

In summary, the practical implications of our review suggest that team sport athletes can benefit from lower training volumes in strength and aerobic-based interventions, if in-field training remains consistent. During tapering phases, athletes may further benefit from reduced training loads without negative effects, potentially enhancing supercompensation. However, caution is needed, and continuous monitoring of adaptations is essential, as the magnitude of training responses and the impact of volume are likely influenced by factors such as competitive level, age, sex, and time of season. Current evidence is still insufficient to provide definitive guidelines in this area.

## Conclusions

The current systematic review with meta-analysis delved into the impact of training volume—ranging from the lower volume to the higher volume—on the physical performance adaptations of team sports players. The predominant focus in the examined studies was on resistance-based training, which incorporates traditional weight-room training, eccentric training, or plyometric training. Relatively few studies concentrated on running-based or combined approaches.

A meta-analysis specifically for resistance-based training within the individual studies revealed a discernible trend. In newly introduced interventions for players, both the lower-volume and higher-volume training volumes (the latter often representing 1.5 to 2.5 times more than the lower-volume) yielded similar effects on the physical performance adaptation of team sports players. Notably, no significant differences were identified between the outcomes. Consequently, given the congested schedules of team sports competitions and the prevalent emphasis on field-based training by coaches, implementing resistance-based training with lower volumes proved effective, facilitating schedule accommodation.

Moreover, reduced training volume during specific phases, notably tapering, was identified across individual studies. A more substantial decrease in load tended to foster improvements in speed, lower limb power, and aerobic performance during the tapering phase.

Despite the limitations inherent in the current systematic review—namely, the inclusion of studies with a very low certainty of evidence due to small sample sizes and a high risk of bias—the available evidence suggests that lower volume training could be advantageous for participants, even in newly introduced interventions or during tapering. Lower-volume yielded effects comparable to those of higher-volume training, making it a more suitable option for busy training schedules or competitive phases of the year. Nevertheless, the ultimate decision must be made by the coach based on an individualized analysis and considering the uniqueness of each athlete through mechanisms of adjustment, encompassing regular assessments and monitoring processes.

## Supplementary Information

Additional file 1.

## Data Availability

All data are available by per request to the corresponding author.

## Abbreviations

COD: Change-of-direction
GRADE: Grading of Recommendations Assessment, Development and Evaluation methodology
PCF: Participant classification framework
RoB 2: Risk of Bias tool, version 2
ROBINS-I: Risk of bias in non-randomized studies of interventions
VO2max: Maximal oxygen uptake
HRmax: Maximal heart rate
