# Supplementary material for: Impact of Lower-Volume Training on Physical Fitness Adaptations in Team Sports Players: A Systematic Review and Meta-analysis
Source: Sports Med Open. 2025 Jan 20;11:3. doi: 10.1186/s40798-024-00808-3 (PMC11747014; doi:10.1186/s40798-024-00808-3)
Supplement: Supplementary file 1 — Additional file 1. [file 40798_2024_808_MOESM1_ESM.docx]

**Supplementary material 1.** Full-text screening.

| **Article** | **Population** | **Intervention** | **Comparator** | **Outcomes** | **Study design** | **Decision** |
| --- | --- | --- | --- | --- | --- | --- |
| Baker, D. G., & Newton, R. U. (2006). Adaptations in upper-body maximal strength and power output resulting from long-term resistance training in experienced strength-power athletes. Journal of Strength and Conditioning Research, 20(3), 541-546. doi:10.1519/R-16024.1 | YES | NO | NO | YES | NO | Exclude |
| Lehnert, M., Janura, M., Jakubec, A., Stejskal, P., & Stelzer, J. (2006). Reaction of Volleyball Players to the Training Microcyle With an Increased Strength Training Volume. International Journal of Volleyball Research, 9(1), 11-18. Retrieved from https://search.ebscohost.com/login.aspx?direct=true&db=s3h&AN=31537684&lang=pt-pt&site=ehost-live&scope=site | YES | NO | NO | YES | NO | Exclude |
| Newton, R. U., Rogers, R. A., Volek, J. S., Häkkinen, K., & Kraemer, W. J. (2006). Four weeks of optimal load ballistic resistance training at the end of season attenuates declining jump performance of women volleyball players. Journal of Strength and Conditioning Research, 20(4), 955-961. doi:10.1519/R-5050502x.1 | YES | NO | NO | YES | NO | Exclude |
| Coutts, A., Reaburn, P., Piva, T. J., & Murphy, A. (2007). Changes in selected biochemical, muscular strength, power, and endurance measures during deliberate overreaching and tapering in rugby league players. International Journal of Sports Medicine, 28(2), 116-124. doi:10.1055/s-2006-924145 | YES | NO | NO | YES | NO | Exclude |
| Coutts, A. J., Reaburn, P., Piva, T. J., & Rowsell, G. J. (2007). Monitoring for overreaching in rugby league players. European Journal of Applied Physiology, 99(3), 313-324. doi:10.1007/s00421-006-0345-z | YES | YES | YES | YES | YES | Include |
| Miller, T. A., Thierry-Aguilera, R., Congleton, J. J., Amendola, A. A., Clark, M. J., Crouse, S. F., . . . Jenkins, O. C. (2007). Seasonal changes in V̇O2max among Division 1A collegiate women soccer players. Journal of Strength and Conditioning Research, 21(1), 48-51. doi:10.1519/00124278-200702000-00009 | YES | NO | NO | YES | NO | Exclude |
| Moore, C. A., & Fry, A. C. (2007). NONFUNCTIONAL OVERREACHING DURING OFF-SEASON TRAINING FOR SKILL POSITION PLAYERS IN COLLEGIATE AMERICAN FOOTBALL. Journal of Strength & Conditioning Research, 21(3), 793-800. Retrieved from https://search.ebscohost.com/login.aspx?direct=true&db=s3h&AN=113579232&lang=pt-pt&site=ehost-live&scope=site | YES | NO | NO | YES | NO | Exclude |
| Rahimi, R. (2007). THE ACUTE EFFECTS OF HEAVY VERSUS LIGHT-LOAD SQUATS ON SPRINT PERFORMANCE. / AKUTNI EFEKAT ČUČNJEVA SA VELIKIM OPETEREĆENJEM U ODNOSU NA ČUČNJEVE SA MALIM OPTEREĆENJEM NA BRZINU TRČANJA U SPRINTU. Facta Universitatis: Series Physical Education & Sport, 5(2), 163-169. Retrieved from https://search.ebscohost.com/login.aspx?direct=true&db=s3h&AN=28086014&lang=pt-pt&site=ehost-live&scope=site | YES | NO | NO | YES | YES | Exclude |
| Ettema, G., Gløsen, T., & van den Tillaar, R. (2008). Effect of Specific Resistance Training on Overarm Throwing Performance. International Journal of Sports Physiology & Performance, 3(2), 164-175. Retrieved from https://search.ebscohost.com/login.aspx?direct=true&db=s3h&AN=33013456&lang=pt-pt&site=ehost-live&scope=site | YES | NO | NO | YES | YES | Exclude |
| Hoffman, J. R., Ratamess, N. A., Klatt, M., Faigenbaum, A. D., Ross, R. E., Tranchina, N. M., . . . Kraemer, W. J. (2009). Comparison between different off-season resistance training programs in division III American college football players. Journal of Strength and Conditioning Research, 23(1), 11-19. doi:10.1519/JSC.0b013e3181876a78 | YES | NO | NO | YES | YES | Exclude |
| Hong-Sun, S., Dong-Ho, P., & Dong-Sik, J. (2009). The Effect of Periodized Strength Training Application on the Korea National Team. International Journal of Applied Sports Sciences, 21(2), 122-145. Retrieved from https://search.ebscohost.com/login.aspx?direct=true&db=s3h&AN=47569152&lang=pt-pt&site=ehost-live&scope=site | NO | NO | NO | YES | NO | Exclude |
| Owen, A. L., & Pui-lam, W. (2009). In-Season Weekly High-Intensity Training Volume Among Professional English Soccer Players: A 20-Week Study. Soccer Journal, 54(2), 28-32. Retrieved from https://search.ebscohost.com/login.aspx?direct=true&db=s3h&AN=36906167&lang=pt-pt&site=ehost-live&scope=site | YES | NO | NO | NO | NO | Exclude |
| Argus, C. K., Gill, N., Keogh, J., Hopkins, W. G., & Beaven, C. M. (2010). Effects of a short-term pre-season training programme on the body composition and anaerobic performance of professional rugby union players. Journal of Sports Sciences, 28(6), 679-686. doi:10.1080/02640411003645695 | YES | NO | NO | YES | NO | Exclude |
| Chelly, M. S., Ghenem, M. A., Abid, K., Hermassi, S., Tabka, Z., & Shephard, R. J. (2010). Effects of in-season short-term plyometric training program on leg power, jump-and sprint performance of soccer players. Journal of Strength and Conditioning Research, 24(10), 2670-2676. doi:10.1519/JSC.0b013e3181e2728f | YES | NO | NO | YES | NO | Exclude |
| McLean, B. D., Coutts, A. J., Kelly, V., McGuigan, M. R., & Cormack, S. J. (2010). Neuromuscular, Endocrine, and Perceptual Fatigue Responses During Different Length Between-Match Microcycles in Professional Rugby League Players. International Journal of Sports Physiology & Performance, 5(3), 367-383. Retrieved from https://search.ebscohost.com/login.aspx?direct=true&db=s3h&AN=55089273&lang=pt-pt&site=ehost-live&scope=site | YES | NO | NO | YES | NO | Exclude |
| Molacek, Z. D., Conley, D. S., Evetovich, T. K., & Hinnerichs, K. R. (2010). Effects of low- and high-volume stretching on bench press performance in collegiate football players. Journal of Strength & Conditioning Research, 24(3), 711-716. Retrieved from https://search.ebscohost.com/login.aspx?direct=true&db=s3h&AN=105140719&lang=pt-pt&site=ehost-live&scope=site | YES | NO | NO | NO | NO | Exclude |
| Mujika, I. (2010). Intense training: the key to optimal performance before and during the taper. Scandinavian Journal of Medicine & Science in Sports, 20, 24-31. Retrieved from https://search.ebscohost.com/login.aspx?direct=true&db=s3h&AN=53474881&lang=pt-pt&site=ehost-live&scope=site | NO | NO | NO | NO | NO | Exclude |
| Mujika, I., Chaouachi, A., & Chamari, K. (2010). Precompetition taper and nutritional strategies: special reference to training during Ramadan intermittent fast. British Journal of Sports Medicine, 44(7), 495-501. doi:10.1136/bjsm.2009.071274 | NO | NO | NO | NO | NO | Exclude |
| Randers, M. B., Nielsen, J. J., Krustrup, B. R., Sundstrup, E., Jakobsen, M. D., Nybo, L., . . . Krustrup, P. (2010). Positive performance and health effects of a football training program over 12 weeks can be maintained over a 1-year period with reduced training frequency. Scandinavian Journal of Medicine & Science in Sports, 20, 80-89. Retrieved from https://search.ebscohost.com/login.aspx?direct=true&db=s3h&AN=48977287&lang=pt-pt&site=ehost-live&scope=site | NO | YES | YES | YES | YES | Exclude |
| Sperlich, B., Eder, F., Broich, H., Krüger, M., Zinner, C., & Mester, J. (2010). Comparison of high-intensity interval training vs. high-volume training in the preparatory phase in under 14-year-old soccer players. Schweizerische Zeitschrift fur Sportmedizin und Sporttraumatologie, 58(4), 120-124. Retrieved from https://www.scopus.com/inward/record.uri?eid=2-s2.0-78751523129&partnerID=40&md5=07774e1b4e87fc056182341b4217f3f6 | YES | NO | NO | YES | YES | Exclude |
| Augustsson, S., Augustsson, J., Thomeé, R., Karlsson, J., Eriksson, B., & Svantesson, U. (2011). Performance enhancement following a strength and injury prevention program: A 26-week individualized and supervised intervention in adolescent female volleyball players. International Journal of Sports Science and Coaching, 6(3), 399-417. doi:10.1260/1747-9541.6.3.399 | NO | NO | NO | YES | YES | Exclude |
| Aziz, A. R., Chia, M., Singh, R., & Wahid, M. F. (2011). Effects of Ramadan Fasting on Perceived Exercise Intensity During High-Intensity Interval Training in Elite Youth Soccer Players. International Journal of Sports Science & Coaching, 6(1), 87-98. Retrieved from https://search.ebscohost.com/login.aspx?direct=true&db=s3h&AN=59345404&lang=pt-pt&site=ehost-live&scope=site | YES | NO | NO | YES | NO | Exclude |
| Chtourou, H., Hammouda, O., Souissi, H., Chamari, K., Chaouachi, A., & Souissi, N. (2011). The Effect of Ramadan Fasting on Physical Performances, Mood State and Perceived Exertion in Young Footballers. Asian Journal of Sports Medicine, 2(3), 177-185. Retrieved from https://search.ebscohost.com/login.aspx?direct=true&db=s3h&AN=76247213&lang=pt-pt&site=ehost-live&scope=site | YES | NO | NO | YES | NO | Exclude |
| Głowacki, A., Ignatiuk, W., Konieczna, A., & Jastrzębski, Z. (2011). Training Load Structure of Young Soccer Players in a Typical Training Microcycle during the Competitive and the Transition Period. Baltic Journal of Health & Physical Activity, 3(1), 26-33. Retrieved from https://search.ebscohost.com/login.aspx?direct=true&db=s3h&AN=73509829&lang=pt-pt&site=ehost-live&scope=site | YES | NO | NO | NO | NO | Exclude |
| Mirzaei, B., & Barjaste, A. (2011). Acute neuromuscular fatigue and metabolic demands in response to two different heavy resistive loading patterns. Medicina dello Sport, 64(2), 125-136. Retrieved from https://www.scopus.com/inward/record.uri?eid=2-s2.0-80052452429&partnerID=40&md5=8cb72d21980110ad78f92a259aad1885 | YES | NO | NO | NO | NO | Exclude |
| Nunes, J. A., Crewther, B. T., Viveiros, L., De Rose Jr, D., & Aoki, M. S. (2011). Effects of resistance training periodization on performance and salivary immune-endocrine responses of elite female basketball players. Journal of Sports Medicine and Physical Fitness, 51(4), 676-682. Retrieved from https://www.scopus.com/inward/record.uri?eid=2-s2.0-84856859735&partnerID=40&md5=70fbabb99c1c42966943662c0bb196a2 | YES | NO | NO | YES | NO | Exclude |
| Sperlich, B., De Marées, M., Koehler, K., Linville, J., Holmberg, H. C., & Mester, J. (2011). Effects of 5 weeks of high-intensity interval training vs. volume training in 14-year-old soccer players. Journal of Strength and Conditioning Research, 25(5), 1271-1278. doi:10.1519/JSC.0b013e3181d67c38 | YES | NO | NO | YES | YES | Exclude |
| Andrejić, O., Tošić, S., & Knežević, O. (2012). ACUTE EFFECTS OF LOW- AND HIGH-VOLUME STRETCHING ON FITNESS PERFORMANCE IN YOUNG BASKETBALL PLAYERS. Serbian Journal of Sports Sciences(1), 11-16. Retrieved from https://search.ebscohost.com/login.aspx?direct=true&db=s3h&AN=78304658&lang=pt-pt&site=ehost-live&scope=site | YES | NO | NO | NO | NO | Exclude |
| Mallo, J. (2012). Effect of block periodization on physical fitness during a competitive soccer season. International Journal of Performance Analysis in Sport, 12(1), 64-74. doi:10.1080/24748668.2012.11868583 | YES | NO | NO | YES | NO | Exclude |
| Manna, I., Khanna, G. L., & Dhara, P. C. (2012). Effect of training on anthropometric, physiological and biochemical variables of U-19 volleyball players. Journal of Human Sport and Exercise, 7(1), 263-274. doi:10.4100/jhse.2012.71.05 | YES | NO | NO | YES | NO | Exclude |
| Meckel, Y., Gefen, Y., Nemet, D., & Eliakim, A. (2012). Influence of short vs. long repetition sprint training on selected fitness components in young soccer players. Journal of Strength and Conditioning Research, 26(7), 1845-1851. doi:10.1519/JSC.0b013e318236d0f0 | YES | NO | NO | YES | YES | Exclude |
| Rowan, A. E., Kueffner, T. E., & Stavrianeas, S. (2012). Short Duration High-Intensity Interval Training Improves Aerobic Conditioning of Female College Soccer Players. International Journal of Exercise Science, 5(3), 232-238. Retrieved from https://search.ebscohost.com/login.aspx?direct=true&db=s3h&AN=82217727&lang=pt-pt&site=ehost-live&scope=site | YES | NO | NO | YES | YES | Exclude |
| Cadore, E. L., Pinheiro, E., Izquierdo, M., Correa, C. S., Radaelli, R., Martins, J. B., . . . Pinto, R. S. (2013). Neuromuscular, hormonal, and metabolic responses to different plyometric training volumes in rugby players. J Strength Cond Res, 27(11), 3001-3010. doi:10.1519/JSC.0b013e31828c32de | YES | NO | NO | YES | NO | Exclude |
| Charalampos, P., Zisis, P., Asterios, P., & Nikolaos, M. (2013). Comparison of two physical conditioning programs in improving aerobic endurance in moderately trained youth amateur soccer players during the preparation period. Journal of Physical Education & Sport, 13(3), 419-424. Retrieved from https://search.ebscohost.com/login.aspx?direct=true&db=s3h&AN=99030579&lang=pt-pt&site=ehost-live&scope=site | YES | NO | NO | YES | YES | Exclude |
| Chen, Z. R., Wang, Y. H., Peng, H. T., Yu, C. F., & Wang, M. H. (2013). The acute effect of drop jump protocols with different volumes and recovery time on countermovement jump performance. Journal of Strength and Conditioning Research, 27(1), 154-158. doi:10.1519/JSC.0b013e3182518407 | YES | NO | NO | YES | NO | Exclude |
| Cook, C. J., Beaven, C. M., & Kilduff, L. P. (2013). Three weeks of eccentric training combined with overspeed exercises enhances power and running speed performance gains in trained athletes. Journal of Strength and Conditioning Research, 27(5), 1280-1286. doi:10.1519/JSC.0b013e3182679278 | YES | NO | NO | YES | YES | Exclude |
| Crewther, B. T., Heke, T., & Keogh, J. W. L. (2013). The effects of a resistance-training program on strength, body composition and baseline hormones in male athletes training concurrently for rugby union 7's. Journal of Sports Medicine and Physical Fitness, 53(1), 34-41. Retrieved from https://www.scopus.com/inward/record.uri?eid=2-s2.0-84877595567&partnerID=40&md5=5ff2faa6fe33bc988852b9eadb1cdbdf | YES | NO | NO | YES | NO | Exclude |
| Faudea, O., Rotha, R., Di Giovinea, D., Zahnera, L., & Donatha, L. (2013). Combined strength and power training in high-level amateur football during the competitive season: a randomised-controlled trial. Journal of Sports Sciences, 31(13), 1460-1467. Retrieved from https://search.ebscohost.com/login.aspx?direct=true&db=s3h&AN=93664659&lang=pt-pt&site=ehost-live&scope=site | YES | NO | NO | YES | NO | Exclude |
| Ingebrigtsen, J., Shalfawi, S. A. I., Tønnessen, E., Krustrup, P., & Holtermann, A. (2013). Performance effects of 6 weeks of aerobic production training in junior elite soccer players. Journal of Strength and Conditioning Research, 27(7), 1861-1867. doi:10.1519/JSC.0b013e31827647bd | YES | NO | NO | YES | NO | Exclude |
| Khazhal Kakahama, S. (2013). EFFECT OF COMPLEX TRAINING WITH LOW-INTENSITY LOADING INTERVAL ON CERTAIN PHYSICAL VARIABLES AMONG VOLLEYBALL INFANTS (10-12 AGES). Ovidius University Annals, Series Physical Education & Sport/Science, Movement & Health, 13(1), 16-21. Retrieved from https://search.ebscohost.com/login.aspx?direct=true&db=s3h&AN=85380185&lang=pt-pt&site=ehost-live&scope=site | YES | NO | NO | YES | NO | Exclude |
| Naclerio, F., Faigenbaum, A., Larumbe, E., Goss-Sampson, M., Perez-Bilbao, T., Jimenez, A., & Beedie, C. (2013). Effects of a Low Volume Injury Prevention Program on the Hamstring Torque Angle Relationship. Research in Sports Medicine, 21(3), 253-263. Retrieved from https://search.ebscohost.com/login.aspx?direct=true&db=s3h&AN=88290499&lang=pt-pt&site=ehost-live&scope=site | YES | NO | NO | YES | NO | Exclude |
| Naclerio, F., Faigenbaum, A. D., Larumbe-Zabala, E., Perez-Bibao, T., Kang, J., Ratamess, N. A., & Triplett, N. T. (2013). Effects of different resistance training volumes on strength and power in team sport athletes. Journal of Strength and Conditioning Research, 27(7), 1832-1840. doi:10.1519/JSC.0b013e3182736d10 | YES | YES | YES | YES | YES | Include |
| Sperlich, B., Hoppe, M. W., & Haegele, M. (2013). Endurance exercise - High volume vs. high-intensity interval training in soccer. Deutsche Zeitschrift fur Sportmedizin, 64(1), 10-17. doi:10.5960/dzsm.2012.052 | YES | NO | NO | NO | NO | Exclude |
| Váczi, M., Tollár, J., Meszler, B., Juhász, I., & Karsai, I. (2013). Short-Term High Intensity Plyometric Training Program Improves Strength, Power and Agility in Male Soccer Players. Journal of Human Kinetics, 37, 17-26. Retrieved from https://search.ebscohost.com/login.aspx?direct=true&db=s3h&AN=86721932&lang=pt-pt&site=ehost-live&scope=site | YES | NO | NO | YES | NO | Exclude |
| Brito, J., Vasconcellos, F., Oliveira, J., Krustrup, P., & Rebelo, A. (2014). Short-Term Performance Effects of Three Different Low-Volume Strength-Training Programmes in College Male Soccer Players. Journal of Human Kinetics, 40, 121-128. Retrieved from https://search.ebscohost.com/login.aspx?direct=true&db=s3h&AN=95758592&lang=pt-pt&site=ehost-live&scope=site | YES | YES | NO | YES | NO | Exclude |
| De Lacey, J., Brughelli, M., McGuigan, M., Hansen, K., Samozino, P., & Morin, J. B. (2014). The effects of tapering on power-forcevelocity profiling and jump performance in professional Rugby League players. Journal of Strength and Conditioning Research, 28(12), 3567-3570. doi:10.1519/JSC.0000000000000572 | YES | NO | NO | YES | NO | Exclude |
| Freitas, C. G., Aoki, M. S., Franciscon, C. A., Arruda, A. F. S., Carling, C., & Moreira, A. (2014). Psychophysiological Responses to Overloading and Tapering Phases in Elite Young Soccer Players. Pediatric Exercise Science, 26(2), 195-202. Retrieved from https://search.ebscohost.com/login.aspx?direct=true&db=s3h&AN=96342656&lang=pt-pt&site=ehost-live&scope=site | YES | NO | NO | YES | NO | Exclude |
| RamÃ­rez-Campillo, R., Meylan, C. s., ÁLvarez, C., HenríQuez-OlguÃ­n, C., MartÃ­nez, C., CaÃ±as-Jamett, R., . . . Izquierdo, M. (2014). EFFECTS OF IN-SEASON LOW-VOLUME HIGH-INTENSITY PLYOMETRIC TRAINING ON EXPLOSIVE ACTIONS AND ENDURANCE OF YOUNG SOCCER PLAYERS. Journal of Strength & Conditioning Research, 28(5), 1335-1342. Retrieved from https://search.ebscohost.com/login.aspx?direct=true&db=s3h&AN=107850408&lang=pt-pt&site=ehost-live&scope=site | YES | NO | NO | YES | NO | Exclude |
| Rebaï, H., Chtourou, H., Zarrouk, N., Harzallah, A., Kanoun, I., Dogui, M., . . . Tabka, Z. (2014). Reducing Resistance Training Volume during Ramadan Improves Muscle Strength and Power in Football Players. International Journal of Sports Medicine, 35(5), 432-437. Retrieved from https://search.ebscohost.com/login.aspx?direct=true&db=s3h&AN=95797844&lang=pt-pt&site=ehost-live&scope=site | YES | YES | YES | YES | YES | Include |
| Attene, G., Laffaye, G., Chaouachi, A., Pizzolato, F., Migliaccio, G. M., & Padulo, J. (2015). Repeated sprint ability in young basketball players: one vs. two changes of direction (Part 2). Journal of Sports Sciences, 33(15), 1553-1563. Retrieved from https://search.ebscohost.com/login.aspx?direct=true&db=s3h&AN=103309598&lang=pt-pt&site=ehost-live&scope=site | YES | NO | NO | YES | YES | Exclude |
| Bartolomei, S., Stout, J. R., Fukuda, D. H., Hoffman, J. R., & Merni, F. (2015). BLOCK VS. WEEKLY UNDULATING PERIODIZED RESISTANCE TRAINING PROGRAMS IN WOMEN. Journal of Strength and Conditioning Research, 29(10), 2679-2687. doi:10.1519/JSC.0000000000000948 | NO | NO | NO | NO | NO | Exclude |
| de Hoyo, M., Pozzo, M., Sañudo, B., Carrasco, L., Gonzalo-Skok, O., Domínguez-Cobo, S., & Morán-Camacho, E. (2015). Effects of a 10-Week In-Season Eccentric-Overload Training Program on Muscle-Injury Prevention and Performance in Junior Elite Soccer Players. International Journal of Sports Physiology & Performance, 10(1), 46-52. Retrieved from https://search.ebscohost.com/login.aspx?direct=true&db=s3h&AN=100184527&lang=pt-pt&site=ehost-live&scope=site | YES | NO | NO | YES | YES | Exclude |
| Franco-Marquez, F., Rodriguez-Rosell, D., Gonzalez-Suarez, J. M., Pareja-Blanco, F., Mora-Custodio, R., Yanez-Garcia, J. M., & Gonzalez-Badillo, J. (2015). Effects of Combined Resistance Training and Plyometrics on Physical Performance in Young Soccer Players. International Journal of Sports Medicine, 36(11), 906-914. doi:10.1055/s-0035-1548890 | YES | NO | NO | YES | YES | Exclude |
| Iaia, F. M., Fiorenza, M., Perri, E., Alberti, G., Millet, G. P., & Bangsbo, J. (2015). The effect of two speed endurance training regimes on performance of soccer players. PLoS ONE, 10(9). doi:10.1371/journal.pone.0138096 | YES | NO | NO | YES | YES | Exclude |
| Loturco, I., Nakamura, F. Y., Kobal, R., Gil, S., Cal Abad, C. C., Cuniyochi, R., . . . Roschel, H. (2015). Training for power and speed: Effects of increasing or decreasing jump squat velocity in elite young soccer players. Journal of Strength and Conditioning Research, 29(10), 2771-2779. doi:10.1519/JSC.0000000000000951 | YES | NO | NO | YES | YES | Exclude |
| Macpherson, T. W., & Weston, M. (2015). The Effect of Low-Volume Sprint Interval Training on the Development and Subsequent Maintenance of Aerobic Fitness in Soccer Players. International Journal of Sports Physiology & Performance, 10(3), 332-338. Retrieved from https://search.ebscohost.com/login.aspx?direct=true&db=s3h&AN=101756191&lang=pt-pt&site=ehost-live&scope=site | YES | NO | NO | YES | MAYBE | Exclude |
| Moreira, A., Kempton, T., Saldanha Aoki, M., Sirotic, A. C., & Coutts, A. J. (2015). The Impact of 3 Different-Length Between-Matches Microcycles on Training Loads in Professional Rugby League Players. International Journal of Sports Physiology & Performance, 10(6), 767-773. Retrieved from https://search.ebscohost.com/login.aspx?direct=true&db=s3h&AN=109127865&lang=pt-pt&site=ehost-live&scope=site | YES | NO | NO | NO | NO | Exclude |
| Naclerio, F., Chapman, M., Larumbe-Zabala, E., Massey, B., Neil, A., & Triplett, T. N. (2015). Effects of Three Different Conditioning Activity Volumes on the Optimal Recovery Time for Potentiation in College Athletes. Journal of Strength and Conditioning Research, 29(9), 2579-2585. doi:10.1519/JSC.0000000000000915 | YES | NO | NO | YES | NO | Exclude |
| Naclerio, F., Larumbe-Zabala, E., Monajati, A., & Goss-Sampson, M. (2015). Effects of two different injury prevention resistance exercise protocols on the hamstring torque-angle relationship: a randomized controlled trial. Research in Sports Medicine, 23(4), 379-393. Retrieved from https://search.ebscohost.com/login.aspx?direct=true&db=s3h&AN=111871324&lang=pt-pt&site=ehost-live&scope=site | YES | NO | NO | YES | YES | Exclude |
| Naimo, M. A., De Souza, E. O., Wilson, J. M., Carpenter, A. L., Gilchrist, P., Lowery, R. P., . . . Joy, J. (2015). High-intensity interval training has positive effects on performance in ice hockey players. International Journal of Sports Medicine, 36(1), 61-66. doi:10.1055/s-0034-1382054 | YES | NO | NO | YES | YES | Exclude |
| Nedrehagen, E. S., & Saeterbakken, A. H. (2015). The Effects of in-Season Repeated Sprint Training Compared to Regular Soccer Training. Journal of Human Kinetics, 49(1), 237-244. Retrieved from https://search.ebscohost.com/login.aspx?direct=true&db=s3h&AN=112319106&lang=pt-pt&site=ehost-live&scope=site | YES | NO | NO | YES | YES | Exclude |
| Papadakis, L., Patras, K., & Georgoulis, A. D. (2015). IN-SEASON CONCURRENT AEROBIC ENDURANCE AND CMJ IMPROVEMENTS ARE FEASIBLE FOR BOTH STARTERS AND NON-STARTERS IN PROFESSIONAL SOCCER PLAYERS: A CASE STUDY. Journal of Australian Strength & Conditioning, 23(5), 19-30. Retrieved from https://search.ebscohost.com/login.aspx?direct=true&db=s3h&AN=112780422&lang=pt-pt&site=ehost-live&scope=site | YES | NO | NO | YES | NO | Exclude |
| Poomsalood, S., & Pakulanon, S. (2015). Effects of 4-week plyometric training on speed, agility, and leg muscle power in male university basketball players: A pilot study. Kasetsart Journal - Social Sciences, 36(3), 598-606. Retrieved from https://www.scopus.com/inward/record.uri?eid=2-s2.0-84954320320&partnerID=40&md5=069cca8ebb833079e8dd4961355e385c | YES | NO | NO | YES | NO | Exclude |
| Ramírez-Campillo, R., Henríquez-Olguín, C., Burgos, C., Andrade, D. C., Zapata, D., Martínez, C., . . . Izquierdo, M. (2015). Effect of Progressive Volume-Based Overload during Plyometric Training on Explosive and Endurance Performance in Young Soccer Players. Journal of Strength and Conditioning Research, 29(7), 1884-1893. doi:10.1519/JSC.0000000000000836 | YES | YES | YES | YES | YES | Include |
| Ramírez-Campillo, R., Meylan, C. M. P., Álvarez-Lepín, C., Henriquez-Olguín, C., Martinez, C., Andrade, D. C., . . . Izquierdo, M. (2015). The effects of interday rest on adaptation to 6 weeks of plyometric training in young soccer players. Journal of Strength and Conditioning Research, 29(4), 972-979. doi:10.1519/JSC.0000000000000283 | YES | NO | NO | YES | YES | Exclude |
| Crewther, B. T., Heke, T., & Keogh, J. (2016). The effects of two equal-volume training protocols upon strength, body composition and salivary hormones in male rugby union players. Biol Sport, 33(2), 111-116. doi:10.5604/20831862.1196511 | YES | NO | NO | YES | YES | Exclude |
| de Sousa Fortes, L., Macedo Vianna, J., dos Santos Silva, D. M., de Gouvêa, M. A., & Serpeloni Cyrino, E. (2016). Effects of tapering on maximum aerobic power in indoor soccer players. / Efeitos do polimento na potência aeróbia máxima em atletas de indoor soccer. Brazilian Journal of Kineanthropometry & Human Performance, 18(3), 341-352. Retrieved from https://search.ebscohost.com/login.aspx?direct=true&db=s3h&AN=117004173&lang=pt-pt&site=ehost-live&scope=site | YES | YES | YES | YES | YES | Include |
| Fessi, M. S., Zarrouk, N., Di Salvo, V., Filetti, C., Barker, A. R., & Moalla, W. (2016). Effects of tapering on physical match activities in professional soccer players. J Sports Sci, 34(24), 2189-2194. doi:10.1080/02640414.2016.1171891 | YES | NO | NO | YES | NO | Exclude |
| Gonzalo-Skok, O., Tous-Fajardo, J., Arjol-Serrano, J. L., Suarez-Arrones, L., Casajús, J. A., & Mendez-Villanueva, A. (2016). Improvement of Repeated-Sprint Ability and Horizontal-Jumping Performance in Elite Young Basketball Players With Low-Volume Repeated-Maximal-Power Training. International Journal of Sports Physiology & Performance, 11(4), 464-473. Retrieved from https://search.ebscohost.com/login.aspx?direct=true&db=s3h&AN=115359884&lang=pt-pt&site=ehost-live&scope=site | YES | NO | NO | YES | NO | Exclude |
| Kageta, T., Tsuchiya, Y., Morishima, T., Hasegawa, Y., Sasaki, H., & Goto, K. (2016). Influences of increased training volume on exercise performance, physiological and psychological parameters. Journal of Sports Medicine and Physical Fitness, 56(7-8), 913-921. | NO | NO | NO | YES | NO | Exclude |
| Kazem, K., Reza, H. M., Mohsen, D., & Alireza, H. K. (2016). The effect of undulating periodized plyometric training on power, sprint, and agility performance. Gazzetta Medica Italiana Archivio per le Scienze Mediche, 175(12), 499-507. Retrieved from https://www.scopus.com/inward/record.uri?eid=2-s2.0-84990829889&partnerID=40&md5=a9e5ff1bf96e0d03166a1d4174ba64c9 | YES | NO | NO | YES | YES | Exclude |
| Mohr, M., & Krustrup, P. (2016). Comparison between two types of anaerobic speed endurance training in competitive soccer players. Journal of Human Kinetics, 51(1), 183-192. Retrieved from https://search.ebscohost.com/login.aspx?direct=true&db=s3h&AN=118669236&lang=pt-pt&site=ehost-live&scope=site | YES | NO | NO | YES | YES | Exclude |
| Otağ, A., Hazar, M., Otağ, İ., & Beyleroğlu, M. (2016). Effect of increasing maximal aerobic exercise on serum gonadal hormones and alpha-fetoprotein in the luteal phase of professional female soccer players. Journal of Physical Therapy Science, 28(3), 807-810. doi:10.1589/jpts.28.807 | YES | NO | NO | NO | NO | Exclude |
| Rosas, F., Ramirez-Campillo, R., Diaz, D., Abad-Colil, F., Martinez-Salazar, C., Caniuqueo, A., . . . Izquierdo, M. (2016). Jump Training in Youth Soccer Players: Effects of Haltere Type Handheld Loading. International Journal of Sports Medicine, 37(13), 1060-1065. doi:10.1055/s-0042-111046 | YES | NO | NO | YES | YES | Exclude |
| Sánchez, J. S., Familiar, C. H., Muñoz, V. M., García, A. G., Fernández, A. R., & González, M. C. (2016). Effect of intermittent training with and without direction changes on the physical performance of young players. Retos(30), 70-75. Retrieved from https://www.scopus.com/inward/record.uri?eid=2-s2.0-84978745552&partnerID=40&md5=48d5387b17e21b3b0808f0c8b1f17a2d | YES | NO | NO | YES | YES | Exclude |
| Taylor, J. M., Macpherson, T. W., McLaren, S. J., Spears, I., & Weston, M. (2016). Two Weeks of Repeated-Sprint Training in Soccer: To Turn or Not to Turn? International Journal of Sports Physiology & Performance, 11(8), 998-1004. Retrieved from https://search.ebscohost.com/login.aspx?direct=true&db=s3h&AN=120516372&lang=pt-pt&site=ehost-live&scope=site | YES | NO | NO | YES | YES | Exclude |
| Wallenta, C., Granacher, U., Lesinski, M., Schünemann, C., & Mühlbauer, T. (2016). Effects of Complex Versus Block Strength Training on the Athletic Performance of Elite Youth Soccer Players. Sportverletzung-Sportschaden, 30(1), 31-37. doi:10.1055/s-0041-106949 | YES | NO | NO | YES | YES | Exclude |
| Yanci, J., Los Arcos, A., Camara, J., Castillo, D., García, A., & Castagna, C. (2016). Effects of horizontal plyometric training volume on soccer players’ performance. Research in Sports Medicine, 24(4), 308-319. Retrieved from https://search.ebscohost.com/login.aspx?direct=true&db=s3h&AN=119140559&lang=pt-pt&site=ehost-live&scope=site | YES | YES | YES | YES | YES | Include |
| Aoki, M. S., Arruda, A. F. S., Freitas, C. G., Miloski, B., Marcelino, P. R., Drago, G., . . . Moreira, A. (2017). Monitoring training loads, mood states, and jump performance over two periodized training mesocycles in elite young volleyball players. International Journal of Sports Science & Coaching, 12(1), 130-137. Retrieved from https://search.ebscohost.com/login.aspx?direct=true&db=s3h&AN=120994276&lang=pt-pt&site=ehost-live&scope=site | YES | NO | NO | NO | NO | Exclude |
| Chaabene, H., & Negra, Y. (2017). The effect of plyometric training volume on athletic performance in prepubertal male soccer players. International Journal of Sports Physiology and Performance, 12(9), 1205-1211. doi:10.1123/ijspp.2016-0372 | YES | YES | YES | YES | YES | Include |
| Chtara, M., Rouissi, M., Haddad, M., Chtara, H., Chaalali, A., Owen, A., & Chamari, K. (2017). Specific physical trainability in elite young soccer players: efficiency over 6 weeks' in-season training. Biology of Sport, 34(2), 137-148. Retrieved from https://search.ebscohost.com/login.aspx?direct=true&db=s3h&AN=123119445&lang=pt-pt&site=ehost-live&scope=site | YES | NO | NO | YES | YES | Exclude |
| Funch, L. T., Lind, E., True, L., Langen, D. V., Foley, J. T., & Hokanson, J. F. (2017). Four weeks of off-season training improves peak oxygen consumption in female field hockey players. Sports, 5(4). doi:10.3390/sports5040089 | YES | NO | NO | YES | YES | Exclude |
| Gonzalo-Skok, O., Tous-Fajardo, J., Valero-Campo, C., Berzosa, C., Bataller, A. V., Arjol-Serrano, J. L., . . . Mendez-Villanueva, A. (2017). Eccentric-Overload Training in Team-Sport Functional Performance: Constant Bilateral Vertical Versus Variable Unilateral Multidirectional Movements. International Journal of Sports Physiology & Performance, 12(7), 951-958. Retrieved from https://search.ebscohost.com/login.aspx?direct=true&db=s3h&AN=125553408&lang=pt-pt&site=ehost-live&scope=site | YES | NO | NO | YES | YES | Exclude |
| Inness, M. W. H., Billaut, F., & Aughey, R. J. (2017). Live-high train-low improves repeated time-trial and Yo-Yo IR2 performance in sub-elite team-sport athletes. Journal of Science and Medicine in Sport, 20(2), 190-195. doi:10.1016/j.jsams.2015.12.518 | YES | NO | NO | YES | YES | Exclude |
| Leceaga, J., Los Arcos, A., Castillo, D., & Yanci, J. (2017). INFLUENCE OF PLYOMETRIC TRAINING VOLUME ON DIFFERENTIATED PERCEIVED EXERTION LOAD OF HIGH-LEVEL SOCCER PLAYERS. PENSAR EN MOVIMIENTO-REVISTA DE CIENCIAS DEL EJERCICIO Y LA SALUD, 15(2). doi:10.15517/pensarmov.v15i2.27664 | YES | YES | YES | NO | YES | Exclude |
| Maroto-Izquierdo, S., García-López, D., & de Paz, J. A. (2017). Functional and Muscle-Size Effects of Flywheel Resistance Training with Eccentric-Overload in Professional Handball Players. Journal of Human Kinetics, 60(1), 133-143. Retrieved from https://search.ebscohost.com/login.aspx?direct=true&db=s3h&AN=127058319&lang=pt-pt&site=ehost-live&scope=site | YES | NO | NO | YES | YES | Exclude |
| Marrier, B., Robineau, J., Piscione, J., Lacome, M., Peeters, A., Hausswirth, C., . . . Le Meur, Y. (2017). Supercompensation Kinetics of Physical Qualities During a Taper in Team-Sport Athletes. International Journal of Sports Physiology & Performance, 12(9), 1163-1169. Retrieved from https://search.ebscohost.com/login.aspx?direct=true&db=s3h&AN=126616080&lang=pt-pt&site=ehost-live&scope=site | YES | NO | NO | YES | NO | Exclude |
| Moran, J., Sandercock, G. R. H., Ramírez-Campillo, R., Todd, O., Collison, J., & Parry, D. A. (2017). Maturation-Related Effect of Low-Dose Plyometric Training on Performance in Youth Hockey Players. Pediatric Exercise Science, 29(2), 194-202. Retrieved from https://search.ebscohost.com/login.aspx?direct=true&db=s3h&AN=122738510&lang=pt-pt&site=ehost-live&scope=site | YES | NO | NO | YES | NO | Exclude |
| Otero-Esquina, C., de Hoyo Lora, M., Gonzalo-Skok, Ó., Domínguez-Cobo, S., & Sánchez, H. (2017). Is strength-training frequency a key factor to develop performance adaptations in young elite soccer players? European Journal of Sport Science, 17(10), 1241-1251. Retrieved from https://search.ebscohost.com/login.aspx?direct=true&db=s3h&AN=125880833&lang=pt-pt&site=ehost-live&scope=site | YES | YES | YES | YES | YES | Include |
| RodrÍGuez-Rosell, D., Franco-MÁRquez, F., Mora-Custodio, R., & GonzÁLez-Badillo, J. J. (2017). EFFECT OF HIGH-SPEED STRENGTH TRAINING ON PHYSICAL PERFORMANCE IN YOUNG SOCCER PLAYERS OF DIFFERENT AGES. Journal of Strength & Conditioning Research, 31(9), 2498-2508. Retrieved from https://search.ebscohost.com/login.aspx?direct=true&db=s3h&AN=125283176&lang=pt-pt&site=ehost-live&scope=site | YES | NO | NO | YES | NO | Exclude |
| Rodríguez-Rosell, D., Torres-Torrelo, J., Franco-Márquez, F., González-Suárez, J. M., & González-Badillo, J. J. (2017). Effects of light-load maximal lifting velocity weight training vs. combined weight training and plyometrics on sprint, vertical jump and strength performance in adult soccer players. Journal of Science & Medicine in Sport, 20(7), 695-699. Retrieved from https://search.ebscohost.com/login.aspx?direct=true&db=s3h&AN=123268366&lang=pt-pt&site=ehost-live&scope=site | YES | NO | NO | YES | YES | Exclude |
| Sabido, R., Hernández-Davó, J. L., Botella, J., Navarro, A., & Tous-Fajardo, J. (2017). Effects of adding a weekly eccentric-overload training session on strength and athletic performance in team-handball players. European Journal of Sport Science, 17(5), 530-538. Retrieved from https://search.ebscohost.com/login.aspx?direct=true&db=s3h&AN=122428506&lang=pt-pt&site=ehost-live&scope=site | YES | NO | NO | YES | NO | Exclude |
| Thuwakum, W., Hamlin, M. J., Manimmanakorn, N., Leelayuwat, N., Wonnabussapawich, P., Boobpachat, D., & Manimmanakorn, A. (2017). Low-load resistance training with hypoxia mimics traditional strength training in team sport athletes. Journal of Physical Education & Sport, 17(1), 240-247. Retrieved from https://search.ebscohost.com/login.aspx?direct=true&db=s3h&AN=123822874&lang=pt-pt&site=ehost-live&scope=site | YES | NO | NO | YES | YES | Exclude |
| Torres-Torrelo, J., Rodríguez-Rosell, D., & González-Badillo, J. J. (2017). Light-load maximal lifting velocity full squat training program improves important physical and skill characteristics in futsal players. Journal of Sports Sciences, 35(10), 967-975. Retrieved from https://search.ebscohost.com/login.aspx?direct=true&db=s3h&AN=121304057&lang=pt-pt&site=ehost-live&scope=site | YES | NO | NO | YES | NO | Exclude |
| Varley, I., Hughes, D. C., Greeves, J. P., Fraser, W. D., & Sale, C. (2017). Increased Training Volume Improves Bone Density and Cortical Area in Adolescent Football Players. International Journal of Sports Medicine, 38(5), 341-346. Retrieved from https://search.ebscohost.com/login.aspx?direct=true&db=s3h&AN=122971476&lang=pt-pt&site=ehost-live&scope=site | YES | NO | NO | NO | NO | Exclude |
| Yanci, J., Castillo, D., Iturricastillo, A., Ayarra, R., & Nakamura, F. Y. (2017). Effects of Two Different Volume-Equated Weekly Distributed Short-Term Plyometric Training Programs on Futsal Players' Physical Performance. Journal of Strength and Conditioning Research, 31(7), 1787-1794. doi:10.1519/JSC.0000000000001644 | YES | NO | NO | YES | YES | Exclude |
| Arazi, H., Khanmohammadi, A., Asadi, A., & Haff, G. G. (2018). The effect of resistance training set configuration on strength, power, and hormonal adaptation in female volleyball players. Applied Physiology, Nutrition & Metabolism, 43(2), 154-164. Retrieved from https://search.ebscohost.com/login.aspx?direct=true&db=s3h&AN=127615131&lang=pt-pt&site=ehost-live&scope=site | YES | NO | NO | YES | YES | Exclude |
| Assuncao, A. R., Bottaro, M., Cardoso, E. A., da Silva, D. P. D., Ferraz, M., Vieira, C. A., & Gentil, P. (2018). Effects of a low-volume plyometric training in anaerobic performance of adolescent athletes. Journal of Sports Medicine and Physical Fitness, 58(5), 570-575. doi:10.23736/S0022-4707.17.07173-0 | YES | NO | NO | YES | NO | Exclude |
| Bazyler, C. D., Mizuguchi, S., Sole, C. J., Suchomel, T. J., Sato, K., Kavanaugh, A. A., . . . Stone, M. H. (2018). Jumping performance is preserved but not muscle thickness in collegiate volleyball players after a taper. Journal of Strength and Conditioning Research, 32(4), 1020-1028. doi:10.1519/JSC.0000000000001912 | YES | NO | NO | YES | NO | Exclude |
| Brownlee, T. E., O’Boyle, A., Morgans, R., Morton, J. P., Erskine, R. M., & Drust, B. (2018). Training duration may not be a predisposing factor in potential maladaptations in talent development programmes that promote early specialisation in elite youth soccer. International Journal of Sports Science & Coaching, 13(5), 674-678. Retrieved from https://search.ebscohost.com/login.aspx?direct=true&db=s3h&AN=132294750&lang=pt-pt&site=ehost-live&scope=site | YES | NO | NO | NO | NO | Exclude |
| Ferioli, D., Bosio, A., Bilsborough, J. C., La Torre, A., Tornaghi, M., & Rampinini, E. (2018). The Preparation Period in Basketball: Training Load and Neuromuscular Adaptations. International Journal of Sports Physiology & Performance, 13(8), 991-999. Retrieved from https://search.ebscohost.com/login.aspx?direct=true&db=s3h&AN=132097649&lang=pt-pt&site=ehost-live&scope=site | YES | NO | NO | NO | NO | Exclude |
| Ferioli, D., Bosio, A., La Torre, A., Carlomagno, D., Connolly, D. R., & Rampinini, E. (2018). DIFFERENT TRAINING LOADS PARTIALLY INFLUENCE PHYSIOLOGICAL RESPONSES TO THE PREPARATION PERIOD IN BASKETBALL. Journal of Strength and Conditioning Research, 32(3), 790-797. doi:10.1519/JSC.0000000000001823 | YES | NO | NO | NO | NO | Exclude |
| Fransson, D., Nielsen, T. S., Olsson, K., Christensson, T., Bradley, P. S., Fatouros, I. G., . . . Mohr, M. (2018). Skeletal muscle and performance adaptations to high-intensity training in elite male soccer players: speed endurance runs versus small-sided game training. European Journal of Applied Physiology, 118(1), 111-121. doi:10.1007/s00421-017-3751-5 | YES | NO | NO | YES | YES | Exclude |
| Gentilcore, D. R. (2018). THE MINIMAL EFFECTIVE DOSE OF NORDIC HAMSTRING EXERCISE FOR REDUCING HAMSTRING STRAIN INJURIES IN FOOTBALL (SOCCER). Journal of Australian Strength & Conditioning, 26(5), 80-87. Retrieved from https://search.ebscohost.com/login.aspx?direct=true&db=s3h&AN=135052312&lang=pt-pt&site=ehost-live&scope=site | YES | NO | NO | NO | NO | Exclude |
| Kelly, D. T., Tobin, C., Egan, B., McCarren, A., OʼConnor, P. L., McCaffrey, N., & Moyna, N. M. (2018). Comparison of sprint interval and endurance training in team sport athletes. Journal of Strength and Conditioning Research, 32(11), 3051-3058. doi:10.1519/JSC.0000000000002374 | YES | NO | NO | YES | YES | Exclude |
| Makhlouf, I., Chaouachi, A., Chaouachi, M., Othman, A. B., Granacher, U., & Behm, D. G. (2018). Combination of agility and plyometric training provides similar training benefits as combined balance and plyometric training in young soccer players. Frontiers in Physiology, 9(NOV). doi:10.3389/fphys.2018.01611 | YES | NO | NO | YES | YES | Exclude |
| Manchado, C., Cortell-Tormo, J. M., & Tortosa-Martínez, J. (2018). Effects of two different training periodization models on physical and physiological aspects of elite female team handball players. Journal of Strength and Conditioning Research, 32(1), 280-287. doi:10.1519/JSC.0000000000002259 | YES | NO | NO | YES | NO | Exclude |
| Mazurek, K., Zmijewski, P., Makaruk, H., Mróz, A., Czajkowska, A., Witek, K., . . . Lipińska, P. (2018). Effects of Short-Term Plyometric Training on Physical Performance in Male Handball Players. Journal of Human Kinetics, 63(1), 137-148. Retrieved from https://search.ebscohost.com/login.aspx?direct=true&db=s3h&AN=131995087&lang=pt-pt&site=ehost-live&scope=site | YES | NO | NO | YES | YES | Exclude |
| Nickerson, B. S., Mangine, G. T., Williams, T. D., & Martinez, I. A. (2018). Effect of cluster set warm-up configurations on sprint performance in collegiate male soccer players. Applied Physiology, Nutrition & Metabolism, 43(6), 625-630. Retrieved from https://search.ebscohost.com/login.aspx?direct=true&db=s3h&AN=129897257&lang=pt-pt&site=ehost-live&scope=site | YES | NO | NO | YES | NO | Exclude |
| Petré, H., Löfving, P., & Psilander, N. (2018). The Effect of Two Different Concurrent Training Programs on Strength and Power Gains in Highly-Trained Individuals. Journal of Sports Science & Medicine, 17(2), 167-173. Retrieved from https://search.ebscohost.com/login.aspx?direct=true&db=s3h&AN=129621063&lang=pt-pt&site=ehost-live&scope=site | YES | NO | NO | YES | YES | Exclude |
| Pliauga, V., Lukonaitiene, I., Kamandulis, S., Skurvydas, A., Sakalauskas, R., Scanlan, A. T., . . . Conte, D. (2018). The effect of block and traditional periodization training models on jump and sprint performance in collegiate basketball players. Biology of Sport, 35(4), 373-382. Retrieved from https://search.ebscohost.com/login.aspx?direct=true&db=s3h&AN=133165129&lang=pt-pt&site=ehost-live&scope=site | YES | NO | NO | YES | YES | Exclude |
| Presland, J. D., Timmins, R. G., Bourne, M. N., Williams, M. D., & Opar, D. A. (2018). The effect of Nordic hamstring exercise training volume on biceps femoris long head architectural adaptation. Scandinavian Journal of Medicine & Science in Sports, 28(7), 1775-1783. doi:10.1111/sms.13085 | NO | YES | YES | YES | YES | Exclude |
| Ramirez-Campillo, R., Alvarez, C., García-Pinillos, F., Sanchez-Sanchez, J., Yanci, J., Castillo, D., . . . Izquierdo, M. (2018). Optimal reactive strength index: Is it an accurate variable to optimize plyometric training effects on measures of physical fitness in young soccer players? Journal of Strength and Conditioning Research, 32(4), 885-893. doi:10.1519/jsc.0000000000002467 | YES | NO | NO | YES | YES | Exclude |
| Ramirez-Campillo, R., García-Pinillos, F., García-Ramos, A., Yanci, J., Gentil, P., Chaabene, H., & Granacher, U. (2018). Effects of different plyometric training frequencies on components of physical fitness in amateur female soccer players. Frontiers in Physiology, 9(JUL). doi:10.3389/fphys.2018.00934 | YES | YES | YES | YES | YES | Include |
| Saavedra, J. M., Porgeirsson, S., Kristjansdottir, H., Halldorsson, K., Gudmundsdottir, M. L., & Einarsson, I. P. (2018). Comparison of training volumes in different elite sportspersons according to sex, age, and sport practised. Montenegrin Journal of Sports Science and Medicine, 7(2), 37-42. doi:10.26773/mjssm.180906 | YES | NO | NO | NO | NO | Exclude |
| Sabido, R., Hernández-Davó, J. L., Botella, J., Jiménez-Leiva, A., & Fernández-Fernández, J. (2018). EFFECTS OF BLOCK AND DAILY UNDULATING PERIODIZATION ON NEUROMUSCULAR PERFORMANCE IN YOUNG MALE HANDBALL PLAYERS. Kinesiology, 50(1), 97-103. Retrieved from https://search.ebscohost.com/login.aspx?direct=true&db=s3h&AN=130456892&lang=pt-pt&site=ehost-live&scope=site | YES | NO | NO | YES | YES | Exclude |
| Sabido, R., Hernández-Davó, J. L., & Pereyra-Gerber, G. T. (2018). Influence of Different Inertial Loads on Basic Training Variables During the Flywheel Squat Exercise. International Journal of Sports Physiology & Performance, 13(4), 482-489. Retrieved from https://search.ebscohost.com/login.aspx?direct=true&db=s3h&AN=129945781&lang=pt-pt&site=ehost-live&scope=site | YES | NO | NO | NO | NO | Exclude |
| Suarez-Arrones, L., de Villarreal, E. S., Núñez, F. J., Di Salvo, V., Petri, C., Buccolini, A., . . . Mendez-Villanueva, A. (2018). In-season eccentric-overload training in elite soccer players: Effects on body composition, strength and sprint performance. PLoS ONE, 13(10). doi:10.1371/journal.pone.0205332 | YES | NO | NO | YES | NO | Exclude |
| Torres-Torrelo, J., Rodríguez-Rosell, D., Mora-Custodio, R., Pareja-Blanco, F., Yañez-García, J. M., & González-Badillo, J. J. (2018). Effects of Resistance Training and Combined Training Program on Repeated Sprint Ability in Futsal Players. International Journal of Sports Medicine, 39(7), 517-526. Retrieved from https://search.ebscohost.com/login.aspx?direct=true&db=s3h&AN=130396334&lang=pt-pt&site=ehost-live&scope=site | YES | NO | NO | YES | YES | Exclude |
| Tsoukos, A., Veligekas, P., Brown, L. E., Terzis, G., & Bogdanis, G. C. (2018). DELAYED EFFECTS OF A LOW-VOLUME,POWER-TYPE RESISTANCE EXERCISE SESSION ON EXPLOSIVE PERFORMANCE. Journal of Strength & Conditioning Research, 32(3), 643-650. Retrieved from https://search.ebscohost.com/login.aspx?direct=true&db=s3h&AN=128249710&lang=pt-pt&site=ehost-live&scope=site | YES | NO | NO | YES | NO | Exclude |
| Ullrich, B., Pelzer, T., & Pfeiffer, M. (2018). NEUROMUSCULAR EFFECTS TO 6 WEEKS OF LOADED COUNTERMOVEMENT JUMPING WITH TRADITIONAL AND DAILY UNDULATING PERIODIZATION. Journal of Strength & Conditioning Research, 32(3), 660-674. Retrieved from https://search.ebscohost.com/login.aspx?direct=true&db=s3h&AN=128249712&lang=pt-pt&site=ehost-live&scope=site | YES | NO | NO | YES | YES | Exclude |
| Vitale, J. A., Povìa, V., Vitale, N. D., Bassani, T., Lombardi, G., Giacomelli, L., . . . La Torre, A. (2018). The effect of two different speed endurance training protocols on a multiple shuttle run performance in young elite male soccer players. Research in Sports Medicine, 26(4), 436-449. Retrieved from https://search.ebscohost.com/login.aspx?direct=true&db=s3h&AN=131640205&lang=pt-pt&site=ehost-live&scope=site | YES | NO | NO | NO | YES | Exclude |
| Bianchi, M., Coratella, G., Dello Iacono, A., & Beato, M. (2019). Comparative effects of single vs. double weekly plyometric training sessions on jump, sprint and change of directions abilities of elite youth football players. Journal of Sports Medicine and Physical Fitness, 59(6), 910-915. doi:10.23736/S0022-4707.18.08804-7 | YES | YES | YES | YES | YES | Include |
| Botonis, P. G., Toubekis, A. G., & Platanou, T. I. (2019). Training Loads, Wellness And Performance Before and During Tapering for a Water-Polo Tournament. Journal of Human Kinetics, 66(1), 131-141. Retrieved from https://search.ebscohost.com/login.aspx?direct=true&db=s3h&AN=135775893&lang=pt-pt&site=ehost-live&scope=site | YES | NO | NO | YES | NO | Exclude |
| Coratella, G., Beato, M., Cè, E., Scurati, R., Milanese, C., Schena, F., & Esposito, F. (2019). Effects of in-season enhanced negative work-based vs traditional weight training on change of direction and hamstrings-to-quadriceps ratio in soccer players. Biology of Sport, 36(3), 241-248. Retrieved from https://search.ebscohost.com/login.aspx?direct=true&db=s3h&AN=138303386&lang=pt-pt&site=ehost-live&scope=site | YES | NO | NO | YES | YES | Exclude |
| Figueiredo, D. H., Figueiredo, D. H., Moreira, A., Gonçalves, H. R., & Stanganelli, L. C. R. (2019). Effect of Overload and Tapering on Individual Heart Rate Variability, Stress Tolerance, and Intermittent Running Performance in Soccer Players During a Preseason. J Strength Cond Res, 33(5), 1222-1231. doi:10.1519/jsc.0000000000003127 | YES | NO | NO | NO | NO | Exclude |
| Freitas, T. T., Calleja-González, J., Carlos-Vivas, J., Marín-Cascales, E., & Alcaraz, P. E. (2019). Short-term optimal load training vs a modified complex training in semi-professional basketball players. Journal of Sports Sciences, 37(4), 434-442. doi:10.1080/02640414.2018.1504618 | YES | NO | NO | YES | YES | Exclude |
| Hostrup, M., Gunnarsson, T. P., Fiorenza, M., Mørch, K., Onslev, J., Pedersen, K. M., & Bangsbo, J. (2019). In‐season adaptations to intense intermittent training and sprint interval training in sub‐elite football players. Scandinavian Journal of Medicine & Science in Sports, 29(5), 669-677. Retrieved from https://search.ebscohost.com/login.aspx?direct=true&db=s3h&AN=135820967&lang=pt-pt&site=ehost-live&scope=site | YES | NO | NO | YES | YES | Exclude |
| Lacome, M., Avrillon, S., Cholley, Y., Simpson, B. M., Guilhem, G., & Buchheit, M. (2019). Hamstring Eccentric Strengthening Program: Does Training Volume Matter? Int J Sports Physiol Perform, 1-27. doi:10.1123/ijspp.2018-0947 | YES | YES | YES | YES | YES | Include |
| Marques, D. L., Travassos, B., Sousa, A. C., Gil, M. H., Ribeiro, J. N., & Marques, M. C. (2019). Effects of Low-Moderate Load High-Velocity Resistance Training on Physical Performance of Under-20 Futsal Players. Sports (2075-4663), 7(3), 69-69. Retrieved from https://search.ebscohost.com/login.aspx?direct=true&db=s3h&AN=135684823&lang=pt-pt&site=ehost-live&scope=site | YES | NO | NO | YES | NO | Exclude |
| Muehlbauer, T., Wagner, V., Brueckner, D., Schedler, S., Schwiertz, G., Kiss, R., & Hagen, M. (2019). Effects of a blocked versus an alternated sequence of balance and plyometric training on physical performance in youth soccer players. BMC Sports Science, Medicine & Rehabilitation, 11(1), N.PAG-N.PAG. Retrieved from https://search.ebscohost.com/login.aspx?direct=true&db=s3h&AN=138395640&lang=pt-pt&site=ehost-live&scope=site | YES | NO | NO | YES | YES | Exclude |
| Ortega-Becerra, M., SigÜEnza-Iglesias, J. A., & AsiÁNclemente, J. A. (2019). Effects of 4-week training with balls of different weights on throwing velocity in handball players. Journal of Physical Education & Sport, 19, 344-349. Retrieved from https://search.ebscohost.com/login.aspx?direct=true&db=s3h&AN=135120015&lang=pt-pt&site=ehost-live&scope=site | YES | NO | NO | YES | YES | Exclude |
| Rønnestad, B. R., Øfsteng, S. J., & Ellefsen, S. (2019). Block periodization of strength and endurance training is superior to traditional periodization in ice hockey players. Scandinavian Journal of Medicine & Science in Sports, 29(2), 180-188. Retrieved from https://search.ebscohost.com/login.aspx?direct=true&db=s3h&AN=134148902&lang=pt-pt&site=ehost-live&scope=site | YES | NO | NO | YES | YES | Exclude |
| Runacres, A., Mackintosh, K. A., & McNarry, M. A. (2019). The effect of constant-intensity endurance training and high-intensity interval training on aerobic and anaerobic parameters in youth. Journal of Sports Sciences, 37(21), 2492-2498. Retrieved from https://search.ebscohost.com/login.aspx?direct=true&db=s3h&AN=138615205&lang=pt-pt&site=ehost-live&scope=site | NO | YES | YES | YES | YES | Exclude |
| Sanchez-Sanchez, J., Gonzalo-Skok, O., Carretero, M., Pineda, A., Ramirez-Campillo, R., & Nakamura, F. Y. (2019). Effects of concurrent eccentric overload and high-intensity interval training on team sports players’ performance. Kinesiology, 51(1), 119-126. doi:10.26582/K.51.1.14 | YES | NO | NO | YES | YES | Exclude |
| Teixeira, A. S., Nunes, R. F. H., Yanci, J., Izzicupo, P., Forner Flores, L. J., Romano, J. C., . . . Nakamura, F. Y. (2019). Different Pathways Leading up to the Same Futsal Competition: Individual and Inter-Team Variability in Loading Patterns and Preseason Training Adaptations. Sports (2075-4663), 7(1), 7-1. Retrieved from https://search.ebscohost.com/login.aspx?direct=true&db=s3h&AN=134358427&lang=pt-pt&site=ehost-live&scope=site | YES | NO | NO | YES | NO | Exclude |
| Zghal, F., Colson, S. S., Blain, G., Behm, D. G., Granacher, U., & Chaouachi, A. (2019). Combined resistance and plyometric training is more effective than plyometric training alone for improving physical fitness of pubertal soccer players. Frontiers in Physiology, 10(AUG). doi:10.3389/fphys.2019.01026 | YES | NO | NO | YES | YES | Exclude |
| Beltran-Valls, M. R., Camarero-López, G., Beltran-Garrido, J. V., & Cecilia-Gallego, P. (2020). Effects of a Tapering Period on Physical Condition in Soccer Players. Journal of Strength and Conditioning Research, 34(4), 1086-1092. doi:10.1519/JSC.0000000000002138 | YES | YES | YES | YES | YES | Include |
| Branquinho, L., Ferraz, R., Mendes, P. D., Petricia, J., Serrano, J., & Marques, M. C. (2020). The Effect of an In-Season 8-Week Plyometric Training Programme Followed By a Detraining Period on Explosive Skills in Competitive Junior Soccer Players. Montenegrin Journal of Sports Science & Medicine, 9(1), 33-40. Retrieved from https://search.ebscohost.com/login.aspx?direct=true&db=s3h&AN=141766238&lang=pt-pt&site=ehost-live&scope=site | YES | NO | NO | YES | NO | Exclude |
| Dobbin, N., Highton, J., Moss, S. L., & Twist, C. (2020). The Effects of In-Season, Low-Volume Sprint Interval Training With and Without Sport-Specific Actions on the Physical Characteristics of Elite Academy Rugby League Players. International Journal of Sports Physiology & Performance, 15(5), 705-713. Retrieved from https://search.ebscohost.com/login.aspx?direct=true&db=s3h&AN=142933536&lang=pt-pt&site=ehost-live&scope=site | YES | NO | NO | YES | NO | Exclude |
| Gavanda, S., Geisler, S., Quitmann, O. J., Bauhaus, H., & Schiffer, T. (2020). Three weeks of detraining does not decrease muscle thickness, strength or sport performance in adolescent athletes. International Journal of Exercise Science, 13(6), 633-644. Retrieved from https://www.scopus.com/inward/record.uri?eid=2-s2.0-85089387121&partnerID=40&md5=16ac4b86ecbdbf4cc88e49370b7df5c9 | YES | NO | NO | YES | YES | Exclude |
| Guida Modesto, K. A., Alves de Oliveira, P. F., Gonçalves Fonseca, H., Porto Azevedo, K., Guzzoni, V., Bottaro, M. F., . . . Quagliotti Durigan, J. L. (2020). Russian and Low-Frequency Currents Induced Similar Neuromuscular Adaptations in Soccer Players: A Randomized Controlled Trial. Journal of Sport Rehabilitation, 29(5), 594-601. Retrieved from https://search.ebscohost.com/login.aspx?direct=true&db=s3h&AN=144431322&lang=pt-pt&site=ehost-live&scope=site | YES | NO | NO | YES | NO | Exclude |
| Hertzog, M., Rumpf, M. C., & Hader, K. (2020). RESISTANCE TRAINING STATUS AND EFFECTIVENESS OF LOW-FREQUENCY RESISTANCE TRAINING ON UPPER-BODY STRENGTH AND POWER IN HIGHLY TRAINED SOCCER PLAYERS. Journal of Strength & Conditioning Research, 34(4), 1032-1039. Retrieved from https://search.ebscohost.com/login.aspx?direct=true&db=s3h&AN=143843764&lang=pt-pt&site=ehost-live&scope=site | YES | NO | NO | YES | NO | Exclude |
| Krespi, M., Sporiš, G., & Trajković, N. (2020). Effects of Two Different Tapering Protocols on Fitness and Physical Match Performance in Elite Junior Soccer Players. J Strength Cond Res, 34(6), 1731-1740. doi:10.1519/jsc.0000000000002861 | YES | YES | YES | YES | YES | Include |
| Lukonaitienė, I., Kamandulis, S., Paulauskas, H., Domeika, A., Pliauga, V., Kreivytė, R., . . . Conte, D. (2020). Investigating the workload, readiness and physical performance changes during intensified 3-week preparation periods in female national Under18 and Under20 basketball teams. Journal of Sports Sciences, 38(9), 1018-1025. Retrieved from https://search.ebscohost.com/login.aspx?direct=true&db=s3h&AN=143138263&lang=pt-pt&site=ehost-live&scope=site | YES | NO | NO | YES | NO | Exclude |
| Medeiros, T. M., Ribeiro-Alvares, J. B., Fritsch, C. G., Oliveira, G. S., Severo-Silveira, L., Pappas, E., & Baroni, B. M. (2020). Effect of Weekly Training Frequency With the Nordic Hamstring Exercise on Muscle-Strain Risk Factors in Football Players: A Randomized Trial. International Journal of Sports Physiology & Performance, 15(7), 1026-1033. Retrieved from https://search.ebscohost.com/login.aspx?direct=true&db=s3h&AN=144482472&lang=pt-pt&site=ehost-live&scope=site | YES | NO | NO | YES | YES | Exclude |
| Negra, Y., Chaabene, H., Sammoud, S., Prieske, O., Moran, J., Ramirez-Campillo, R., . . . Granacher, U. (2020). The increased effectiveness of loaded versus unloaded plyometric jump training in improving muscle power, speed, change of direction, and kicking-distance performance in prepubertal male soccer players. International Journal of Sports Physiology and Performance, 15(2), 189-195. doi:10.1123/ijspp.2018-0866 | YES | NO | NO | YES | YES | Exclude |
| Ramirez-Campillo, R., Álvarez, C., García-Pinillos, F., García-Ramos, A., Loturco, I., Chaabene, H., & Granacher, U. (2020). Effects of Combined Surfaces vs. Single-Surface Plyometric Training on Soccer Players' Physical Fitness. Journal of Strength and Conditioning Research, 34(9), 2644-2653. doi:10.1519/JSC.0000000000002929 | YES | NO | NO | YES | YES | Exclude |
| Ramirez-Campillo, R., Alvarez, C., Gentil, P., Loturco, I., Sanchez-Sanchez, J., Izquierdo, M., . . . Granacher, U. (2020). Sequencing Effects of Plyometric Training Applied Before or After Regular Soccer Training on Measures of Physical Fitness in Young Players. Journal of Strength and Conditioning Research, 34(7), 1959-1966. doi:10.1519/JSC.0000000000002525 | YES | NO | NO | YES | YES | Exclude |
| Bern, S., Harris, G., Ramirez-Campillo, R., Chaabene, H., Hammami, R., Rumpf, M. C., & Moran, J. (2021). RESISTANCE AND ENDURANCE TRAINING ARE SIMILARLY EFFECTIVE WHEN DELIVERED IN SEPARATE VERSUS COMBINED FORMATS IN FEMALE RUGBY PLAYERS. Journal of Australian Strength & Conditioning, 29(6), 14-21. Retrieved from https://search.ebscohost.com/login.aspx?direct=true&db=s3h&AN=158131013&lang=pt-pt&site=ehost-live&scope=site | YES | NO | NO | YES | YES | Exclude |
| Cuthbert, M., Haff, G. G., Arent, S. M., Ripley, N., McMahon, J. J., Evans, M., & Comfort, P. (2021). Effects of Variations in Resistance Training Frequency on Strength Development in Well-Trained Populations and Implications for In-Season Athlete Training: A Systematic Review and Meta-analysis. Sports Medicine, 51(9), 1967-1982. Retrieved from https://search.ebscohost.com/login.aspx?direct=true&db=s3h&AN=151899870&lang=pt-pt&site=ehost-live&scope=site | NO | NO | NO | NO | NO | Exclude |
| Dawkins, J., Ishøi, L., Willott, J. O., Andersen, L. L., & Thorborg, K. (2021). Effects of a low-dose Copenhagen adduction exercise intervention on adduction strength in sub-elite male footballers: A randomised controlled trial. Translational Sports Medicine, 4(4), 447-457. doi:10.1002/tsm2.238 | YES | NO | NO | YES | NO | Exclude |
| Ferland, P. M., Garcia, J. E. V., & Comtois, A. S. (2021). Minimal intervention significantly improves agility in female collegiate volleyball players. Gazzetta Medica Italiana Archivio per le Scienze Mediche, 180(4), 114-120. doi:10.23736/S0393-3660.19.04190-1 | YES | NO | NO | YES | NO | Exclude |
| García-Cardona, D., Landázuri, P., & Sánchez-Muñoz, O. (2021). Effect of a shock micro-cycle on biochemical markers in university soccer players. International Journal of Environmental Research and Public Health, 18(7). doi:10.3390/ijerph18073581 | YES | NO | NO | YES | NO | Exclude |
| Kalmus, O. E., Viru, M., Alvar, B., & Naclerio, F. (2021). Impact of low volume velocity-controlled vs. Repetition to failure resistance training session on measures of explosive performance in a team of adolescents basketball players. Sports, 9(8). doi:10.3390/sports9080115 | YES | NO | NO | YES | NO | Exclude |
| Kelly, D. T., Cregg, C. J., O'Connor, P. L., Cullen, B. D., & Moyna, N. M. (2021). Physiological and performance responses of sprint interval training and endurance training in Gaelic football players. European Journal of Applied Physiology, 121(8), 2265-2275. Retrieved from https://search.ebscohost.com/login.aspx?direct=true&db=s3h&AN=151271794&lang=pt-pt&site=ehost-live&scope=site | YES | NO | NO | YES | YES | Exclude |
| Moalla, W., Fessi, M. S., Nouira, S., Mendez-Villanueva, A., Di Salvo, V., & Ahmaidi, S. (2021). Optimal Pretaper Phase on Physical Match Performance in Professional Soccer. International Journal of Sports Physiology & Performance, 16(10), 1483-1489. Retrieved from https://search.ebscohost.com/login.aspx?direct=true&db=s3h&AN=153677486&lang=pt-pt&site=ehost-live&scope=site | YES | NO | NO | NO | NO | Exclude |
| Palma-Muñoz, I., Ramírez-Campillo, R., Azocar-Gallardo, J., Álvarez, C., Asadi, A., Moran, J., & Chaabene, H. (2021). EFFECTS of PROGRESSED and NONPROGRESSED VOLUME-BASED OVERLOAD PLYOMETRIC TRAINING on COMPONENTS of PHYSICAL FITNESS and BODY COMPOSITION VARIABLES in YOUTH MALE BASKETBALL PLAYERS. Journal of Strength and Conditioning Research, 35(6), 1642-1649. doi:10.1519/JSC.0000000000002950 | YES | YES | YES | YES | YES | Include |
| Pereira, A., Teixeira, C., Pereira, K., Ferreira, L., Marques, M., & Silva, A. G. (2021). Neural Mobilization Short-Term Dose Effect on the Lower-Limb Flexibility and Performance in Basketball Athletes: A Randomized, Parallel, and Single-Blinded Study. Journal of Sport Rehabilitation, 30(7), 1060-1066. Retrieved from https://search.ebscohost.com/login.aspx?direct=true&db=s3h&AN=152124571&lang=pt-pt&site=ehost-live&scope=site | YES | YES | YES | NO | YES | Exclude |
| Ramirez-Campillo, R., Moran, J., Drury, B., Williams, M., Keogh, J. W., Chaabene, H., & Granacher, U. (2021). Effects of equal volume but different plyometric jump training intensities on components of physical fitness in physically active young males. Journal of Strength and Conditioning Research, 35(7), 1916-1923. doi:10.1519/JSC.0000000000003057 | YES | NO | NO | YES | YES | Exclude |
| Ramirez-Campillo, R., Pereira, L. A., Andrade, D. C., Mendez-Rebolledo, G., de la Fuente, C. I., Castro-Sepulveda, M., . . . Loturco, I. (2021). Tapering strategies applied to plyometric jump training: A systematic review with meta-analysis of randomized-controlled trials. Journal of Sports Medicine and Physical Fitness, 61(1), 53-62. doi:10.23736/S0022-4707.20.11128-9 | YES | NO | NO | NO | NO | Exclude |
| Rejc, E., Floreani, M., Vaccari, F., Giovanelli, N., Botter, A., Ganzini, A., & Lazzer, S. (2021). Effects of underweight-plyometric training on the neuromuscular characteristics in professional rugby players. Gazzetta Medica Italiana Archivio per le Scienze Mediche, 180(11), 722-729. doi:10.23736/S0393-3660.20.04546-5 | YES | NO | NO | YES | NO | Exclude |
| Severo-Silveira, L., Dornelles, M. P., Lima-E-Silva, F. X., Marchiori, C. L., Medeiros, T. M., Pappas, E., & Baroni, B. M. (2021). Progressive Workload Periodization Maximizes Effects of Nordic Hamstring Exercise on Muscle Injury Risk Factors. Journal of Strength and Conditioning Research, 35(4), 1006-1013. doi:10.1519/JSC.0000000000002849 | YES | YES | YES | YES | YES | Include |
| Vachon, A., Berryman, N., Mujika, I., Paquet, J.-B., & Bosquet, L. (2021). Tapering and Repeated High-Intensity Effort Ability in Young Elite Rugby Union Players: Influence of Pretaper Fatigue Level. International Journal of Sports Physiology & Performance, 16(7), 993-1000. Retrieved from https://search.ebscohost.com/login.aspx?direct=true&db=s3h&AN=151085939&lang=pt-pt&site=ehost-live&scope=site | YES | NO | NO | YES | NO | Exclude |
| Watkins, C. M., Gill, N. D., Maunder, E., Downes, P., Young, J. D., McGuigan, M. R., & Storey, A. G. (2021). The Effect of Low-Volume Preseason Plyometric Training on Force-Velocity Profiles in Semiprofessional Rugby Union Players. Journal of Strength and Conditioning Research, 35(3), 604-615. doi:10.1519/JSC.0000000000003917 | YES | NO | NO | YES | NO | Exclude |
| Abdelkader, M., Hammami, R., Drury, B., Clark, N., Sandercock, G., Shaw, I., . . . Moran, J. (2022). A randomised controlled trial of 1- versus 2-day per week formats of Nordic hamstring training on explosive athletic tasks in prepubertal soccer players. Journal of Sports Sciences, 40(19), 2173-2181. doi:10.1080/02640414.2022.2145737 | YES | NO | NO | YES | NO | Exclude |
| Amundsen, R., Heimland, J. S., Thorarinsdottir, S., Møller, M., & Bahr, R. (2022). Effects of High and Low Training Volume with the Nordic Hamstring Exercise on Hamstring Strength, Jump Height, and Sprint Performance in Female Football Players: A Randomised Trial. Translational Sports Medicine, 1-9. Retrieved from https://search.ebscohost.com/login.aspx?direct=true&db=s3h&AN=158810822&lang=pt-pt&site=ehost-live&scope=site | YES | NO | NO | YES | YES | Exclude |
| Cadu, J. P., Goreau, V., & Lacourpaille, L. (2022). A Very Low Volume of Nordic Hamstring Exercise Increases Maximal Eccentric Strength and Reduces Hamstring Injury Rate in Professional Soccer Players. Journal of Sport Rehabilitation, 31(8), 1061-1066. doi:10.1123/jsr.2021-0445 | YES | NO | NO | YES | NO | Exclude |
| Chena Sinovas, M., Morcillo Losa, J. A., Rodríguez Hernández, M. L., & Zapardiel, J. C. (2022). MULTIVARIATE TRAINING PLANNING MODEL IN PROFESSIONAL SOCCER. Revista Internacional de Medicina y Ciencias de la Actividad Fisica y del Deporte, 22(85), 183-197. doi:10.15366/rimcafd2022.85.012 | YES | NO | NO | YES | NO | Exclude |
| Moran, J., Vali, N., Drury, B., Hammami, R., Tallent, J., Chaabene, H., & Ramirez-Campillo, R. (2022). The effect of volume equated 1- versus 2-day formats of Nordic hamstring exercise training on fitness in youth soccer players: A randomised controlled trial. PLoS ONE, 17(12 December). doi:10.1371/journal.pone.0277437 | YES | NO | NO | YES | NO | Exclude |
| Shah, S., Collins, K., & Macgregor, L. J. (2022). The Influence of Weekly Sprint Volume and Maximal Velocity Exposures on Eccentric Hamstring Strength in Professional Football Players. Sports (2075-4663), 10(8), 125-125. Retrieved from https://search.ebscohost.com/login.aspx?direct=true&db=s3h&AN=158944442&lang=pt-pt&site=ehost-live&scope=site | YES | NO | NO | NO | NO | Exclude |
| Siddle, J., Weaver, K., Greig, M., Harper, D., & Brogden, C. M. (2022). A low-volume Nordic hamstring curl programme improves change of direction ability, despite no architectural, strength or speed adaptations in elite youth soccer players. Research in Sports Medicine. doi:10.1080/15438627.2022.2079984 | YES | NO | NO | YES | NO | Exclude |
| Soto García, D., Díaz Cruz, J., Bautista, I. J., & Martínez Martín, I. (2022). Effects of a Strength Training Protocol with Self-loading and Plyometry on Handball Physical Performance: First National Female Category. E-Balonmano.com: Revista de Ciencias del Deporte, 18(2), 83-92. Retrieved from https://www.scopus.com/inward/record.uri?eid=2-s2.0-85131690718&partnerID=40&md5=8277609a328af7b5e402e20ceeead610 | YES | NO | NO | YES | NO | Exclude |
| Vachon, A., Berryman, N., Mujika, I., Paquet, J.-B., Sauvet, F., & Bosquet, L. (2022). Impact of tapering and proactive recovery on young elite rugby union players' repeated high intensity effort ability. Biology of Sport, 39(3), 735-743. Retrieved from https://search.ebscohost.com/login.aspx?direct=true&db=s3h&AN=158252642&lang=pt-pt&site=ehost-live&scope=site | YES | NO | NO | NO | NO | Exclude |
| Vardakis, L., Michailidis, Y., Mandroukas, A., Zelenitsas, C., Mavrommatis, G., & Metaxas, T. (2022). Effects of a shock microcycle after COVID-19 lockdown period in elite soccer players. Science and Sports, 37(7), 572-580. doi:10.1016/j.scispo.2022.07.004 | YES | NO | NO | YES | NO | Exclude |
| Bonder, I. J., & Shim, A. L. (2023). In-Season Training Model for National Association of Intercollegiate Athletics Female Basketball Players Using "Microdosed" Programming. Strength & Conditioning Journal, 45(4), 395-410. Retrieved from https://search.ebscohost.com/login.aspx?direct=true&db=s3h&AN=169974637&lang=pt-pt&site=ehost-live&scope=site | YES | NO | NO | NO | NO | Exclude |
| Boopathy, D., & Balaji, P. (2023). EFFECT OF DIFFERENT PLYOMETRIC TRAINING VOLUME ON SELECTED MOTOR FITNESS COMPONENTS AND PERFORMANCE ENHANCEMENT OF SOCCER PLAYERS. Ovidius University Annals, Series Physical Education & Sport/Science, Movement & Health, 23(2), 146-153. Retrieved from https://search.ebscohost.com/login.aspx?direct=true&db=s3h&AN=166108697&lang=pt-pt&site=ehost-live&scope=site | YES | NO | NO | YES | NO | Exclude |
| Boraczyński, M. T., Laskin, J. J., Gajewski, J., Podstawski, R. S., Brodnicki, M. A., & Boraczyński, T. W. (2023). Effects of two low-volume high-intensity interval training protocols in professional soccer: sprint interval training versus small-sided games. Journal of Sports Medicine and Physical Fitness, 63(1), 23-33. doi:10.23736/S0022-4707.22.13589-9 | YES | NO | NO | YES | YES | Exclude |
| Byrkjedal, P. T., Thunshelle, A., Spencer, M., Luteberget, L. S., Ivarsson, A., Vårvik, F. T., . . . Bjørnsen, T. (2023). In-season autoregulation of one weekly strength training session maintains physical and external load match performance in professional male football players. Journal of Sports Sciences, 41(6), 536-546. Retrieved from https://search.ebscohost.com/login.aspx?direct=true&db=s3h&AN=164943417&lang=pt-pt&site=ehost-live&scope=site | YES | NO | NO | YES | YES | Exclude |
| Cuadrado-Peñafiel, V., Castaño-Zambudio, A., Martínez-Aranda, L. M., González-Hernández, J. M., Martín-Acero, R., & Jiménez-Reyes, P. (2023). Microdosing Sprint Distribution as an Alternative to Achieve Better Sprint Performance in Field Hockey Players. Sensors, 23(2). doi:10.3390/s23020650 | YES | NO | NO | YES | YES | Exclude |
| Grazioli, R., Inacio, M., Lopez, P., Freitas, S. R., Cunha, G. D., Machado, C. L. F., . . . Pinto, R. S. (2023). Effects of eccentric-emphasized leg curl intervention on muscle strength imbalance markers in professional soccer players during pre-season. Journal of Bodywork and Movement Therapies, 35, 28-32. doi:10.1016/j.jbmt.2023.04.012 | YES | YES | NO | YES | NO | Exclude |
| Grazioli, R., Loturco, I., Lopez, P., Setuain, I., Goulart, J., Veeck, F., . . . Cadore, E. L. (2023). Effects of Moderate-to-Heavy Sled Training Using Different Magnitudes of Velocity Loss in Professional Soccer Players. Journal of Strength and Conditioning Research, 37(3), 629-635. doi:10.1519/JSC.0000000000003813 | YES | No | No | YES | MAYBE | Exclude |
| Krzysztofik, M., Wilk, M., Pisz, A., Kolinger, D., Bichowska, M., Zajac, A., & Stastny, P. (2023). Acute Effects of High-Load vs. Plyometric Conditioning Activity on Jumping Performance and the Muscle-Tendon Mechanical Properties. Journal of Strength & Conditioning Research, 37(7), 1397-1403. Retrieved from https://search.ebscohost.com/login.aspx?direct=true&db=s3h&AN=169981921&lang=pt-pt&site=ehost-live&scope=site | YES | NO | NO | NO | NO | Exclude |
| McQuilliam, S. J., Clark, D. R., Erskine, R. M., & Brownlee, T. E. (2023). Effect of High-Intensity vs. Moderate-Intensity Resistance Training on Strength, Power, and Muscle Soreness in Male Academy Soccer Players. Journal of Strength & Conditioning Research, 37(6), 1250-1258. Retrieved from https://search.ebscohost.com/login.aspx?direct=true&db=s3h&AN=169976970&lang=pt-pt&site=ehost-live&scope=site | YES | NO | NO | YES | MAYBE | Exclude |
| Rathi, A., Sharma, D., & Thapa, R. K. (2023). Effects of complex-descending versus traditional resistance training on physical fitness abilities of female team sports athletes. Biomedical Human Kinetics, 15(1), 148-158. doi:10.2478/bhk-2023-0018 | YES | NO | NO | YES | NO | Exclude |
| Shi, Q., Tong, T. K., Nie, J., Tao, D., Zhang, H., Tan, X., & Kong, Z. (2023). Repeated-sprint training in hypoxia boosts up team-sport-specific repeated-sprint ability: 2-week vs 5-week training regimen. European Journal of Applied Physiology. doi:10.1007/s00421-023-05252-x | YES | YES | YES | YES | YES | Include |

| INCLUDED LATER - MANUAL |  |  |  |  |  |  |
| --- | --- | --- | --- | --- | --- | --- |
| Hoffman, J. R., Kraemer, W. J., Fry, A. C., Deschenes, M., & Kemp, M. (1990). The effects of self-selection for frequency of training in a winter conditioning program for football. The Journal of Strength & Conditioning Research, 4(3), 76-82. | YES | YES | YES | YES | YES | INCLUDED |
